# Tetraphenylanthraquinone and Dihydroxybenzene-Tethered Conjugated Microporous Polymer for Enhanced CO_2_ Uptake and Supercapacitive Energy Storage

**DOI:** 10.1021/jacsau.4c00537

**Published:** 2024-08-16

**Authors:** Mohamed Gamal Mohamed, Chia-Chi Chen, Mervat Ibrahim, Aya Osama Mousa, Mohamed Hammad Elsayed, Yunsheng Ye, Shiao-Wei Kuo

**Affiliations:** † Department of Materials and Optoelectronic Science, Center for Functional Polymers and Supramolecular Materials, National Sun Yat-Sen University, Kaohsiung 804, Taiwan; ‡ Department of Chemistry, Faculty of Science, Assiut University, Assiut 71516, Egypt; § Chemistry Department, Faculty of Science, New Valley University, El-Kharja 72511, Egypt; ∥ Department of Chemistry, Faculty of Science, Al-Azhar University, Nasr City,Cairo 11884, Egypt; ⊥ Department of Medicinal and Applied Chemistry, Kaohsiung Medical University, Kaohsiung 807, Taiwan

**Keywords:** tetraphenylanthraquinone, dihydroxybenzene, conjugated microporous polymers, CO_2_ uptake, supercapacitors

## Abstract

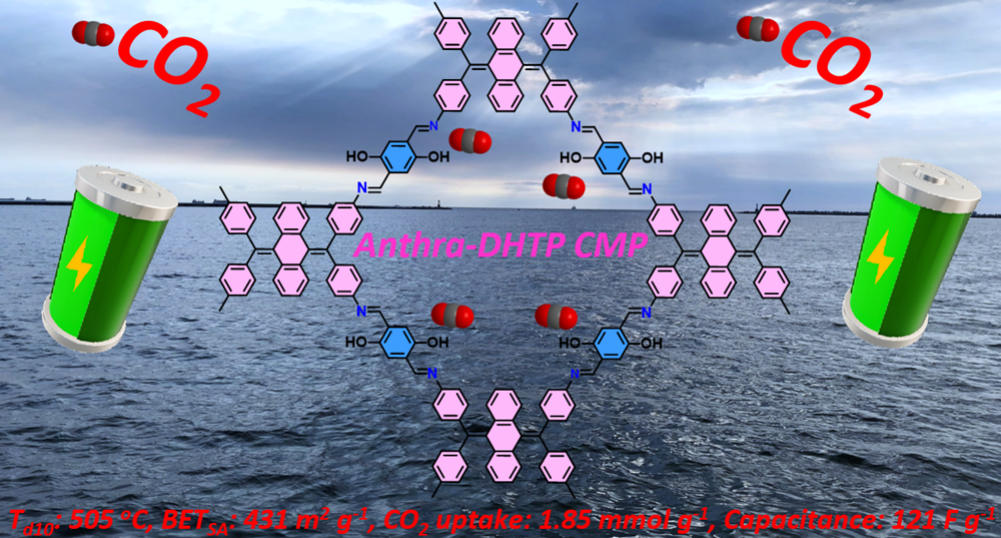

Conjugated microporous polymers (CMPs) feature extended excellent porosity properties and fully conjugated electronic systems, making them highly effective for several uses, including photocatalysis, dye adsorption, CO_2_ capture, supercapacitors, and so on. These polymers are known for their high specific surface area and adjustable porosity. To synthesize DHTP-CMPs (specifically TPE-DHTP CMP and Anthra-DHTP CMP) with abundant nitrogen (N) and oxygen (O) adsorption sites and spherical structures, we employed a straightforward Schiff-base [4 + 2] condensation reaction. This involved using 2,5-dihydroxyterephthalaldehyde (DHTP-2CHO) as the primary building block and phenolic OH group source, along with two distinct structures: 4,4′,4″,4”’-(ethene-1,1,2,2-tetrayl)tetraaniline (TPE-4NH_2_) and 4,4′,4″,4”’-(anthracene-9,10-diylidenebis(methanediylylidene))tetraaniline (Anthra-4Ph-4NH_2_). The synthesized Anthra-DHTP CMP had a remarkable BET surface area (BET_SA_) of 431 m^2^ g^–1^. Additionally, it exhibited outstanding thermal stability, as shown by a *T*_d10_ of 505 °C. Furthermore, for practical implementation, the Anthra-DHTP CMP demonstrates a significant capacity for capturing CO_2_, measuring 1.85 mmol g^–1^ at a temperature of 273 K and 1 bar. In a three-electrode test, the Anthra-DHTP CMP has a remarkable specific capacitance of 121 F g^–1^ at 0.5 A g^–1^. Furthermore, even after undergoing 5000 cycles, it maintains a capacitance retention rate of 79%. Due to their outstanding pore characteristics, abundant N and O, and conjugation properties, this Anthtra-DHTP CMP holds significant potential for CO_2_ capture and supercapacitor applications. This work will pave the way for the development of materials based on DHTP-CMPs and their postmodification with additional groups, facilitating their use in photocatalysis, photodegradation, lithium battery applications, and so on.

## Introduction

The rapid increase in emissions of carbon dioxide (CO_2_) from the burning of fossil fuels presents significant challenges for global societies, including climate change, resource depletion, and elevated levels of environmental contamination.^1−9^ Considerable endeavors are underway to mitigate CO_2_ emissions employing developing affordable, environmentally friendly devices, aimed at fostering enduring advantages for our societies.^10−13^ For instance, electrochemical energy storage (EES) systems like lithium-ion batteries (LIBs), supercapacitors (SCs), and water-splitting electrolyzers, are gaining prominence for energizing developing electronic devices due to their dependable functionality and flexibility.^14−22^ BILs and SCs are essential components of electronically stored energy systems. SCs offer numerous advantages over batteries, including extensive cycle longevity, good power densities, and remarkable rate handling capacities, classifying them as large-capacity energy storage systems.^22−27^ As a result, SCs are valuable for energy storage in industrial, transportation, and technological applications. Additionally, optimizing the power density and performance of supercapacitors necessitates careful consideration of the physical and chemical properties of the electrode materials. Therefore, to meet current demands, a shift toward advanced materials and products is required.^28−30^

Supercapacitors may be classified into three primary categories according to their energy storage techniques: electric double-layer capacitors (EDLCs), battery-like materials, and pseudocapacitive devices.^31^ SCs are widely utilized in various applications such as high-power supplies, multilevel inverters, advanced electric, and vehicles. Their extensive cycle life, high power density, excellent rate capability, wide operating temperature range, ultrafast charge/discharge rates, reversibility, and potential to meet increasing power demands make them a promising solution to address energy scarcity.^32−36^ Despite progress, SCs still have lower energy densities compared to other rechargeable batteries, and their market share in energy storage devices remains relatively small.^37,38^ To expand the applicability of SC devices, significant endeavors are being directed toward augmenting the energy densities of SCs and engineering materials endowed with multifaceted attributes, such as extensive surface areas, customizable porous architectures, highly conductive systems, and enhanced wettability.^39^

Conjugated polymers are often preferred in SCs due to their cost-effectiveness and widespread availability.^40^ For example, electrodes made from polyaniline/composites nanostructures have shown a capacitance of 1221 F g^–1^.^41^ Nonetheless, conventional conjugated polymers (for example, polypyrrole, polyaniline, and polythiophene) are typically devoid of porosity and lack sufficient durability.^42^ Typically, their capacitance declines significantly after more than 1000 charge and discharge cycles. CMPs are a subset of porous organic polymers characterized by extended π-conjugated structures within their microporous networks, showing promising potential for use in supercapacitors.^43,44^ These porous architectures, distinct from those found in linear polymers, are expected to significantly improve electron and ion transport capabilities.^45^ CMPs are traditionally synthesized using various C–N or C–C coupling reactions, such as oxidative polymerization, Buchwald-Hartwig amination, Sonogashira-Hagihara coupling, Schiff base formation, Suzuki-Miyaura coupling, cyclotrimerization, Yamamoto coupling, and phenazine ring fusion methods.^46−50^ These techniques have been utilized to fashion CMPs showcasing varied structural configurations and intrinsic attributes.^51^ CMPs have been extensively studied for their applicability in various fields, including light-emitting diodes, energy conversion, chemosensing, energy storage, catalysis, and various subfields of biological sciences.^52−57^ The suitability of CMPs as active electrode materials for supercapacitors has been assessed by studying CMPs with different structural variations.^58−62^ Due to the diverse building blocks used in CMP structures, their ability to modify π-conjugated units, and their inherent tunability, CMPs often surpass traditional electrode materials in supercapacitor electrode design.^63^ Tetraphenylanthraquinone (Anthra-4Ph) is a chemical molecule with a complex aromatic structure, featuring an anthraquinone core bonded to four phenyl groups. Renowned for its strong chemical stability and unique electrical properties, Anthra-4Ph has garnered interest across a wide range of scientific fields. Its conjugated structure allows for significant electron delocalization, making it a valuable component in organic electronics, photovoltaics, and photochemical applications.^64,65^ Mohamed et al. synthesized An-CPOP-2 using Anthra-4Ph and 2,4,6-trichloro-1,3,5-triazine as monomers. The resulting material demonstrated a CO_2_ capacity of 1.52 mmol g^–1^ (6.7 wt %, 273 K) and capacitance of 98.4 F g^–1^.^64^ Our group-prepared PET-Im CMP through Sonogashira coupling with a specific capacitance of 63 F g^–1^.^43^ According to Bhaumik and co-workers, TFR-NDA demonstrated a capacitance of 362 F g^–1^ in a three-electrode setup.^66^ The Pandey group disclosed that IITR-COF possesses a high specific capacitance of 182.6 F g^–1^, as measured at 0.3 A g^–1^.^67^

In this work, using a straightforward and effective Schiff-base condensation process, we successfully created two types of DHTP-CMPs with CH=N and phenolic units: TPE-DHTP CMP and Anthra-DHTP CMP. Anthra-DHTP CMP is notable for its exceptional thermal stability, with a thermal decomposition temperature (*T*_d10_) of 505 °C and a char yield of 68 wt %. Based on N_2_ adsorption–desorption and CO_2_ isotherms, Anthra-DHTP CMP has the largest BET_SA_ of 431 m^2^ g^–1^ and the highest CO_2_ absorption capacity (1.85 mmol g^–1^ or 8.14 wt % at 273 K). Furthermore, Anthra-DHTP CMP demonstrated capacitance values of 121 F g^–1^ in the GCD tests. This study showcases the efficient production of DHTP-CMPs through free-metal [4 + 2] condensation reaction and highlights their potential applications beyond supercapacitors and CO_2_ absorption. These DHTP-CMP materials are also expected to show promise in related areas such as iodine and dye capture, photocatalysis, and other fields.

## Experimental Section

### Materials

4-Aminophenylboronic acid (BZB-NH_2_, 98%), tin (Sn, ≥99.8%), carbon tetrabromide (CBr_4_, 99%), tetrahydrofuran (THF), potassium carbonate (K_2_CO_3_, ≥99.8%), acetic acid (AcOH, ≥99%), anthraquinone (Anthra, 97%), toluene, triphenylphosphine (PPh_3_, 99%), 4,4′-diaminobenzophenone (BZP-2NH_2_, 97%), 1,4-dioxane (DO), mesitylene (98%), and acetone, tetrakis(triphenylphosphine)palladium [Pd(PPh_3_)_4_, 98%], anhydrous magnesium sulfate (MgSO_4_, ≥99.5%), and sodium hydroxide (NaOH, ≥98%), 2,5-dihydroxyterephthalaldehyde (DHTP-2CHO, 98%), were ordered from Sigma-Aldrich and Alfa Aesar.

### Synthesis of TPE-4NH_2_

BZP-2NH_2_ (3.5 g, 15.73 mmol) was added to 170 mL of HCl at 60 °C. Subsequently, 10.5 g (88 mmol) of Sn was gradually added, and the reaction for 24 at 80 °C. After cooling the flask, the white precipitate was rinsed with NaOH (1 M) solution to acquire a green powder (Scheme S1). FTIR (cm^–1^): 3423, 3359 (N–H), 3030 (aromatic C–H), 1616. ^1^H NMR [DMSO-*d*_6_, δ, ppm, Figure S1]: 6.6 (8H), 6.3 (8H), 4.8 (NH_2_). ^13^C NMR [DMSO-*d*_6_, δ, ppm, Figure S2]: 146–113.1.

### Synthesis of 9,10-Bis(dibromomethylene)-9,10-dihydroanthracene (Anthra-Br_4_)

CBr_4_ (80 g, 303.7 mmol), Anthra (10 g, 48 mmol), and PPh_3_ (50 g, 151 mmol) in a 250 mL two-neck flask. Subsequently, we added 700 mL of toluene to the flask and cooled the mixture to −10 °C using an ice bath and acetone. The reaction mixture was kept for 2 h at −10 °C and then heated to 100 °C. The insoluble material was removed by vacuum filtration following the reaction. The organic layer was evaporated using a rotary evaporator, and the resulting substance was recrystallized from MeOH, yielding a light-yellow solid [6.37 g, Scheme S2]. FTIR (cm^–1^, Figure S3): 3069 (aromatic C–H), 1559. ^1^H NMR [DMSO-*d*_6_, δ, ppm, Figure S4]: 7.8–7.83 (4H), 7.3–7.3 (4H). ^13^C NMR [DMSO-*d*_6_, δ, ppm, Figure S5]: 139.8–91.45.

### Synthesis of Anthra-4Ph-4NH_2_

K_2_CO_3_ (3.2 g, 23.1 mmol), Pd(PPh_3_)_4_ (0.33 g, 0.3 mmol), Anthra-Br_4_ (1.00 g, 2.9 mmol), BZB-NH_2_ (3.2 g, 23.1 mmol), and were mixed in a 250 mL two-neck flask containing a combination of DO and H_2_O (80/40 mL) and increased the temperature reaction to 100 °C for 48 h. The insoluble material was removed by filtration and the reaction solution was poured into HCl (5 mL) with H_2_O (400 mL) resulting in the formation of a light blue precipitate. This solid was then added to 300 mL of MeOH and stirred vigorously for 1 h at 60 °C, yielding Anthra-4Ph-4NH_2_ (2.5 g, Scheme S3). FTIR (cm^–1^): 3429, 3344 (N–H), 3024 (aromatic C–H). ^1^H NMR [DMSO-*d*_6_, δ, ppm, Figure S6]: 7.62, 6.95, 6.69, 6.44, 4.99 (NH_2_). 7.95–6.44. ^13^C NMR [DMSO-*d*_6_, δ, ppm, Figure S7]: 147.9–113.82.

### Preparation of TPE-DHTP CMP and Anthra-DHTP CMP

Use a 25 mL Schlenk tube, 0.10 g of TPE-4NH_2_ (0.25 mmol), 0.08 g of DHTP-2CHO (0.48 mmol), DO/mesitylene (6 mL/6 mL), and 1.5 mL of AcOH (3 M). The solution mixture was refluxed at 110 °C for 3 days. After the reaction, the solid was filtered and purified using Soxhlet extraction [with THF and EtOH; respectively]. Finally, the resulting mixture was dried in a vacuum oven for 1 day, yielding a red powder known as TPE-DHTP CMP. FTIR (KBr, cm^–1^): 3373 (OH stretching), 3029 (C=C–H), 1663 (C=N). Solid-state ^13^C NMR: 147.31 (C=N), 137.89–120.75 ppm (aromatic carbons). For **Anthra-DHTP CMP**: 0.10 g of Anthra-4Ph-4NH_2_ (0.18 mmol), 0.08 g of DHTP-2CHO (0.48 mmol), DO/mesitylene (6 mL/6 mL), and 1.5 mL of AcOH (3 M) to obtain Anthra-DHTP CMP as a brown powder (Yield: 84%) FTIR (cm^–1^): 3372 (OH stretching), 3037 (C=C–H), 1666 (C=N). Solid-state ^13^C NMR: 149 (C=N), 140.12–124.57 ppm (aromatic carbons). As illustrated in Figure S8, the solubility of TPE-DHTP and Anthra-DHTP CMPs was tested in various organic solvents, including acetone, MeOH, DMF, DCM, and THF. The results indicated that both TPE-DHTP and Anthra-DHTP CMPs were insoluble in these solvents.

## Results and Discussion

### Synthesis and Characterization of TPE-DHTP CMP and Anthra-DHTP CMP

**Figure 1:**
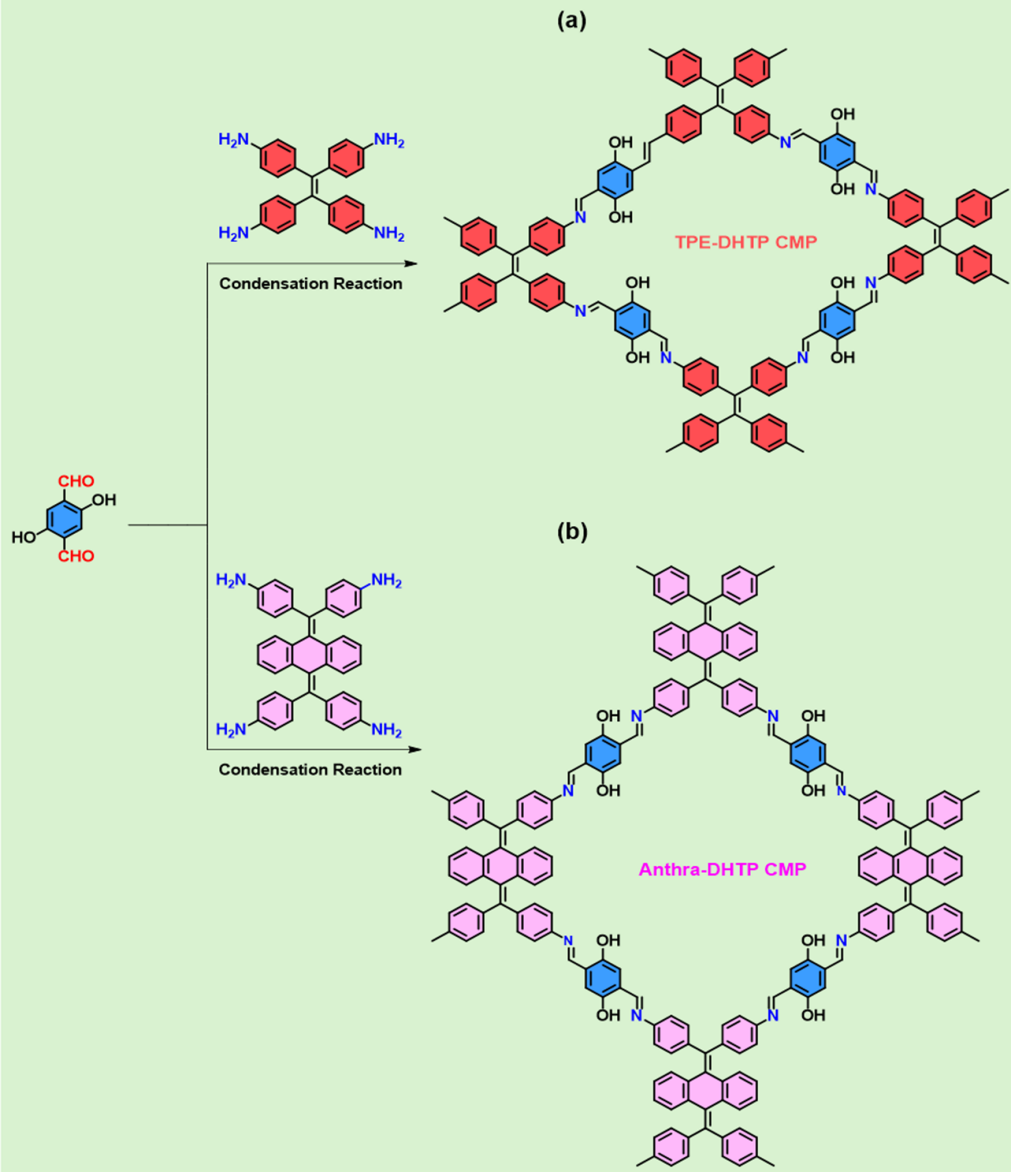
Using a Schiff base reaction for the synthesis of (a) TPE-DHTP and (b) Anthra-DHTP CMPs from DHTP-2CHO as the building block.

**Figure 2:**
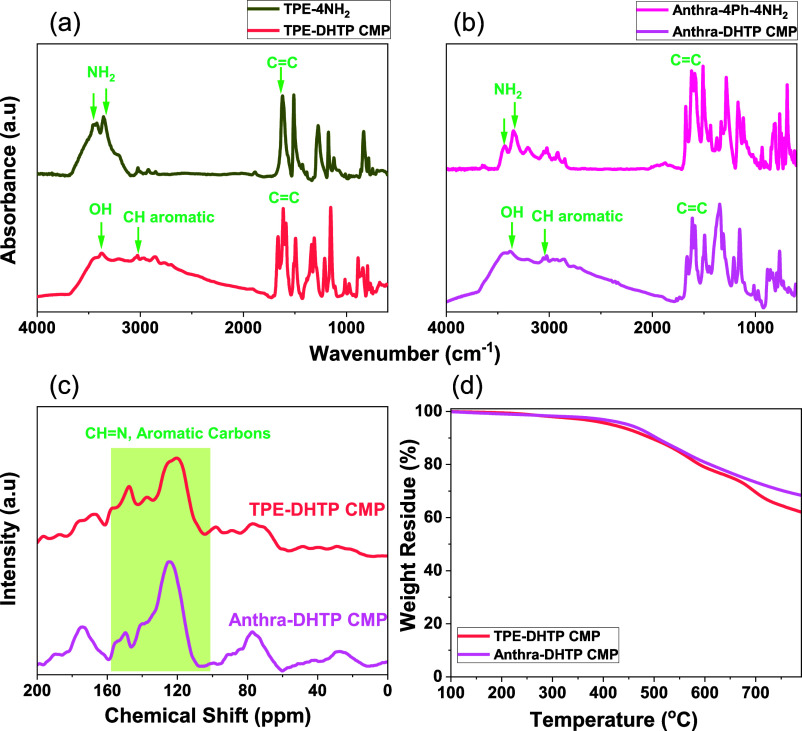
(a) and (b) FTIR spectra of TPE-4NH_2_, TPE-DHTP CMP, Anthra-4Ph-4NH_2_, and Anthra-DHTP CMP. (c) Solid-state ^13^C NMR spectra and (d) TGA traces of TPE-DHTP and Anthra-DHTP CMPs.

We developed TPE-DHTP and Anthra-DHTP-linked CMPs (DHTP-CMPs) enriched with OH and C=N units, leveraging the potential of heteroatoms (N and O) in CMP structures for effective adsorption sites in supercapacitors and CO_2_ capture [through robust physical interactions]. These DHTP-CMPs were synthesized using the Schiff base condensation polymerization technique [4 + 2]. Figure 1a,b illustrate the utilization of DHTP-2CHO [served as the building unit and a source of OH groups in the synthesis process] and TPE-4NH_2_ and Anthra-4Ph-4NH_2_ in DO/mesitylene with AcOH (3 M). Scheme S1 outlines the synthesis of TPE-4NH_2_, which is achieved by reacting BZP-2NH_2_ with Sn in an HCl solution, yielding a green powder. In Scheme S2, Anthra-4Ph-4NH_2_ is prepared by reacting the Anthra unit with CBr_4_/PPh_3_ in toluene to obtain Anthra-Br_4_, a light-yellow powder. Subsequently, Scheme S3 illustrates the Suzuki coupling reaction between Anthra-Br_4_ and BZB-NH_2_ in the presence of Pd(PPh_3_)_4_/K_2_CO_3_ in DO/H_2_O mixtures to produce Anthra-4Ph-4NH_2_. To confirm the synthesis of TPE-4NH_2_, Anthra-4Ph-4NH_2_, TPE-DHTP, and Anthra-DHTP-linked CMPs, FT-IR spectroscopy was initially conducted (Figure 2a,b). Strong absorption peaks corresponding to NH_2_ groups, aromatic CH bonds, and C=C bonds are evident in Figure 2a,b. Specifically, for TPE-NH_2_, peaks were observed at 3423, 3359, 3030, and 1616 cm^–1^, while for Anthra-4Ph-4NH_2_, peaks appeared at 3429, 3344, 3024, and 1612 cm^–1^. The OH and aromatic CH absorption bands in the TPE-DHTP and Anthra-DHTP CMPs were detected between 3378 and 3372 cm^–1^ and 3029 to 3037 cm^–1^, respectively, following the [4 + 2] condensation process involving DTHP-2CHO with TPE-4NH_2_ and Anthra-4Ph-4NH_2_. Furthermore, the absence of the C=O and NH signals in FTIR spectra of DHTP-CMPs indicates the successful construction of the DHTP-CMPs.^61,62^ The structure of both DHTP-CMPs was further analyzed using solid-state ^13^C NMR spectroscopy. In the TPE-DHTP CMP, two distinct resonance peaks were observed: one at 147.31 ppm corresponding to the C=N signal, and another in the range of 137.89–120.75 ppm representing aromatic carbon signals, as depicted in Figure 2c.

**Figure 3:**
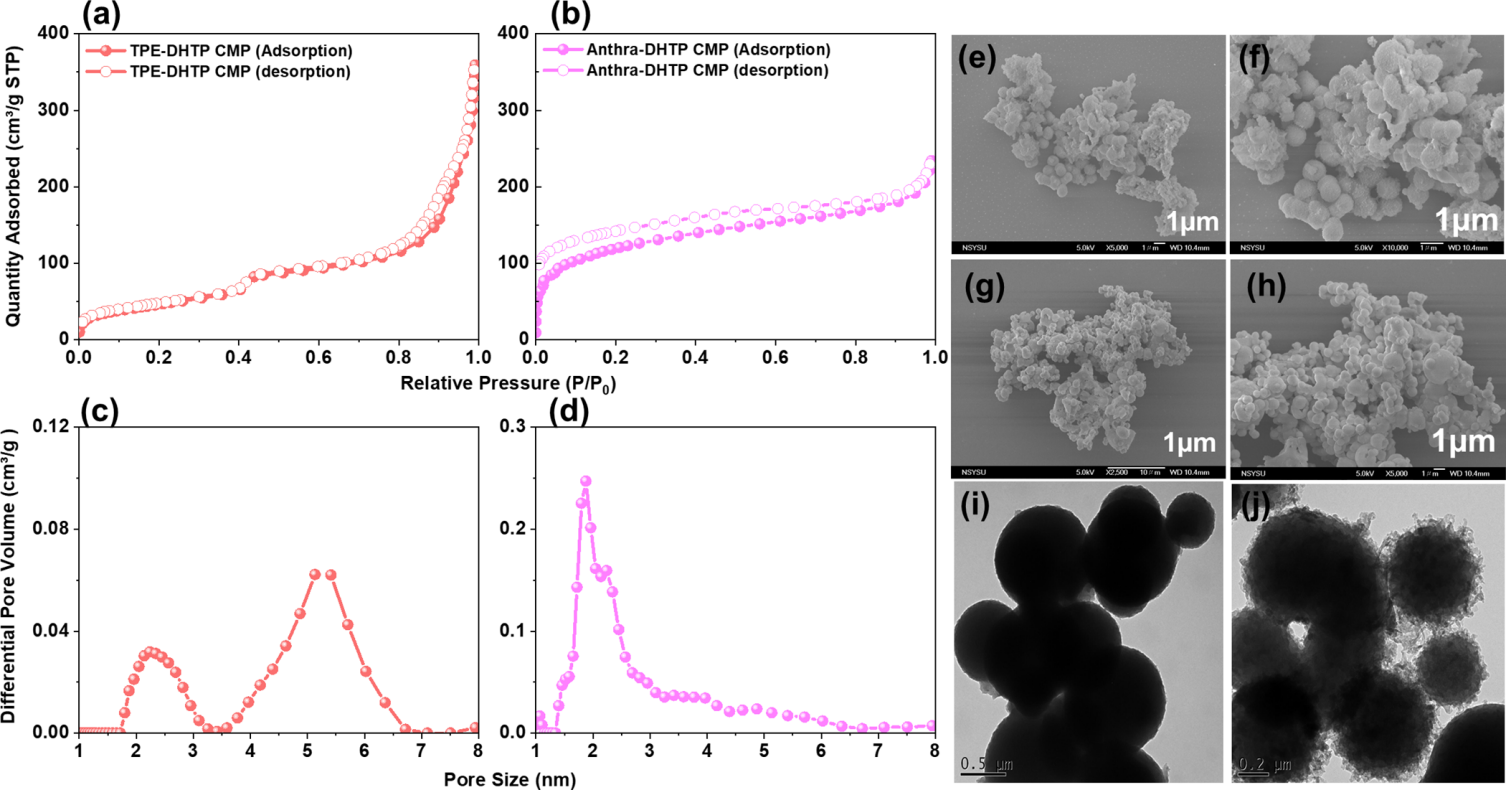
(a and b) N_2_ adsorption–desorption isotherms and (c and d) pore size distributions of (a, c) TPE-DHTP CMP, and (b, d) Anthra-DHTP CMP. (e–h) SEM images and (i, j) TEM images of (e, f, i) TPE-DHTP CMP and (f, h, j) Anthra-DHTP CMP.

Similarly, in the Anthra-DHTP CMP, the C=N signal appeared at 149.0 ppm, while the aromatic carbon signals were observed in the range of 140.12–124.57 ppm. The XPS survey of TPE-DHTP and Anthra-DHTP CMPs, as seen in Figure S9a and S10a, revealed three peaks corresponding to the binding energies of C 1s, N 1s, and O 1s. The chemical states of C, N, and O for TPE-DHTP and Anthra-DHTP CMPs were further analyzed by deconvoluting the high-resolution XPS spectra. The three peaks of high-resolution C 1s XPS spectra deconvolution for TPE-DHTP and Anthra-DHTP CMPs are associated with C=C–C (284.4 eV), C–OH (286.3 eV), and C=N (285.38 eV), as shown in Figures S9b and S10b. Both TPE-DHTP and Anthra-DHTP CMPs displayed one peak for C=N–C bonds at 399.0 and 398.8 eV, respectively, based on the HR N 1s XPS spectral deconvolution, as presented in Figures S9c and S10c. Additionally, the O 1s XPS spectral deconvolution revealed a C–OH peak centered at 532.5 eV for TPE-DHTP CMP and at 532.8 eV for Anthra-DHTP CMP, as illustrated in Figures S9d and S10d. According to the TGA results (Figure 2d), TPE-DHTP CMP exhibited *T*_d5_ and *T*_d10_ values of 417 and 493 °C, respectively. Upon heating to 800 °C, it yielded a char yield of 62 wt %. Similarly, Anthra-DHTP CMP showed *T*_d5_ and *T*_d10_ values of 449 and 505 °C, respectively, with a char yield of 68 wt %. These findings underscore the excellent thermal stability of both DHTP-CMPs. Furthermore, Anthra-DHTP CMP exhibits superior thermal stability compared to TPE-DHTP CMP, likely due to the structural distinctions between the two compounds. The structure of Anthra-DHTP CMP may include stronger intermolecular forces and a more rigid framework, contributing to its higher thermal resistance. We conducted N_2_ adsorption/desorption isotherm measurements at 77 K and utilized SEM and TEM to investigate the morphology (Figure 3), aiming to elucidate the porous characteristics of DHTP-CMPs. Figure 3a,b depict the N_2_ adsorption/desorption isotherm curves. The results indicate that TPE-DHTP CMP exhibits a rapid increase in adsorption at low relative pressures (*P*/*P*_0_ < 0.1), indicative of strong N_2_ interaction. According to the IUPAC classification, this isotherm curve is categorized as Type II. Additionally, Brunauer–Emmett–Teller (BET) analysis revealed a total pore volume (*V*_total_) of 0.53 cm^3^ g^–1^ and a BET_SA_ of 168 m^2^ g^–1^. The pore size distribution reveals two pore sizes at 2.3 and 4.2 nm (Figure 3c). Similarly, Anthra-DHTP CMP exhibits a Type IV isotherm characterized by a rapid increase in adsorption at low relative pressures and the presence of a hysteresis loop. This suggests its microporous nature, supported by BET_SA_ of 431 m^2^ g^–1^, a *V*_total_ of 0.36 cm^3^ g^–1^, and a pore size of 1.9 and 2.3 nm (Figure 3d). The TPE-DHTP-CMP and Anthra-DHTP-CMP exhibit irregular nonspherical particles, as shown by the SEM pictures in Figure 3e–h. The SEM findings were also comparable with TEM images (Figure 3i,j).

**Figure 4:**
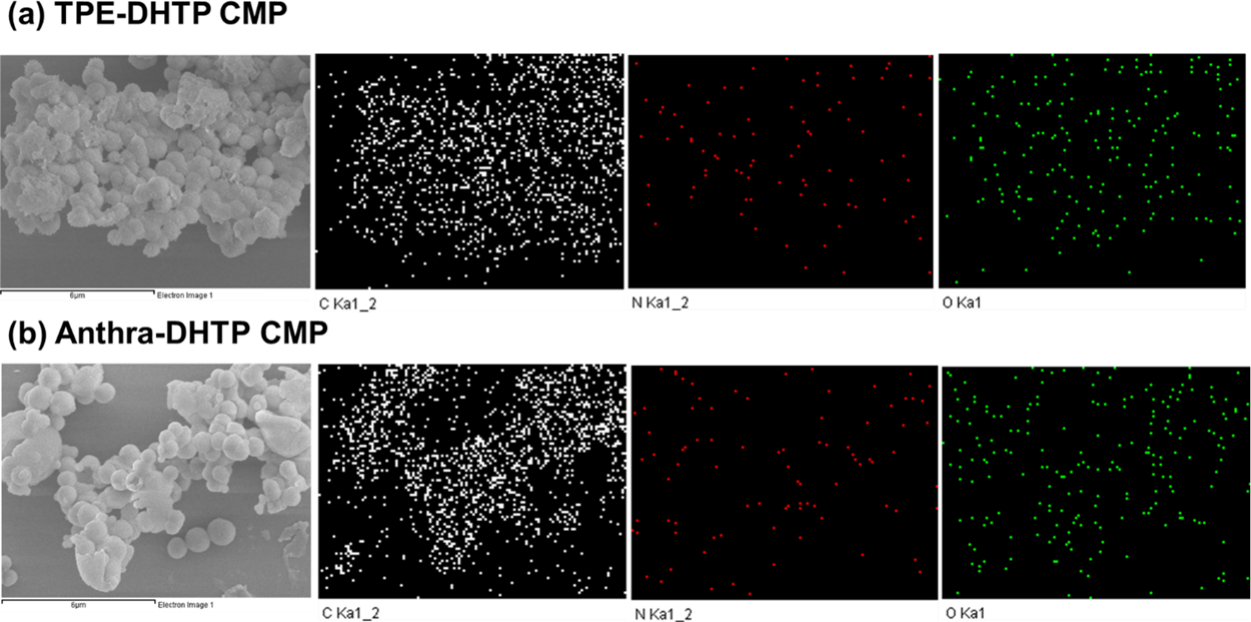
Elemental analyses through SEM-EDS mapping of (a) TPE-DHTP CMP and (b) Anthra-DHTP CMP.

SEM-EDS element mapping images depicted in Figure 4a,b indicate the uniform distribution of elements C (white color), N (red color), and O (green color) throughout the networks of both TPE-DHTP CMP and Anthra-DHTP CMP. Figures S11 and S12 from the XRD study illustrate the amorphous nature of both DHTP-CMP networks, as evidenced by a broad peak.

**Figure 5:**
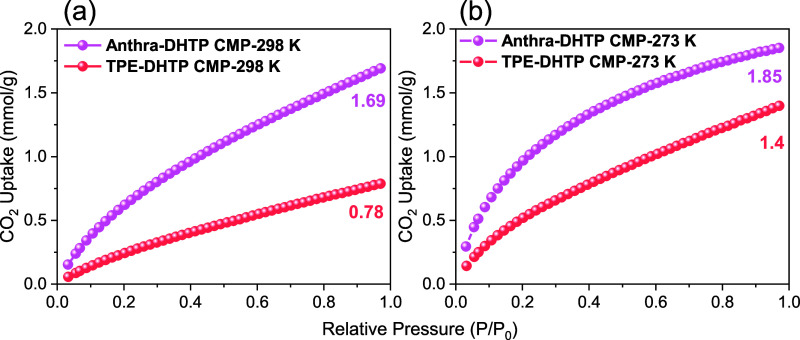
CO_2_ absorption efficiency of the TPE-DHTP and Anthra-DHTP CMPs was evaluated at temperatures of (a) 298 and (b) 273 K.

As seen in Figure 5a,b, further research was conducted on the CO_2_ uptake isotherms at 298 and 273 K for TPE-DHTP CMP and Anthra-DHTP CMP to evaluate their practical adoption. The Anthra-DHTP CMP exhibits a higher capacity for CO_2_ adsorption at both 298 K (1.69 mmol g^–1^, 7.43 wt %) and 273 K (1.85 mmol g^–1^, 8.14 wt %) compared to TPE-DHTP CMP [0.87 (3.83 wt %) and 1.4 (6.16 wt %) mmol g^–1^, respectively measured at 298 and 273 K]. The variation in adsorption can be ascribed to the greater BET_SA_ of Anthra-DHTP CMP and the potential presence of phenolic OH units and N atoms within its framework, which may act as basic sites to capture acidic CO_2_ molecules.^68−70^ The Clausius–Clapeyron equation was used to determine the isosteric heat of adsorption (*Q*_st_) for Anthra-DHTP and TPE-DHTP CMPs. The results revealed that at a low CO_2_ adsorption level of approximately 0.5 mmol/g, the *Q*_st_ for Anthra-DHTP CMP is 29.01 kJ/mol, while for TPE-DHTP CMP, it is 21.46 kJ/mol. These values were derived from the CO_2_ adsorption results obtained at 298 and 273 K. It is worth noting that our DHTP-CMPs demonstrate superior CO_2_ adsorption compared to other porous organic materials. These materials include pyrene-PAFs (0.90–1.15 mmol g^–1^),^71^ CMP-1-(OH)__2__ (1.8 mmol g^–1^),^72^ tri(4-ethynylphenyl)amine-PAFs (1.19 mmol g^–1^),^73^ and CMP-1-AMD1 (1.51 mmol g^–1^).^74^

### Electrochemical Performance of TPE-DHTP CMP and Anthra-DHTP CMP Based on Three Electrodes System

**Figure 6:**
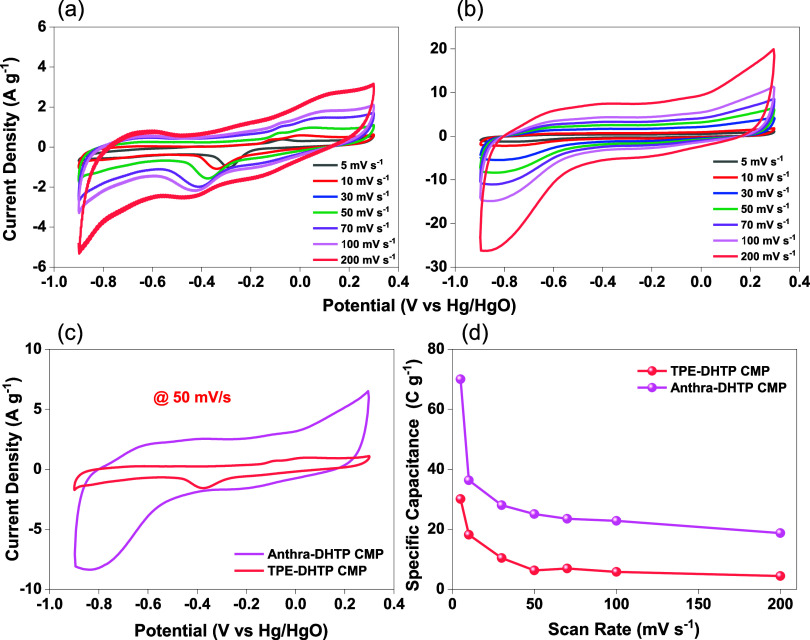
Cyclic voltammetry (CV) curves of (a) TPE-DHTP and (b) Anthra-DHTP CMPs at 5–200 mV s^–1^, (c) CV curve of DHTP-CMPs electrodes were obtained using a scan rate of 50 mV s^–1^ and (d) the relationship between specific capacitance and scan rate for DHTP-CMPs electrodes.

**Figure 7:**
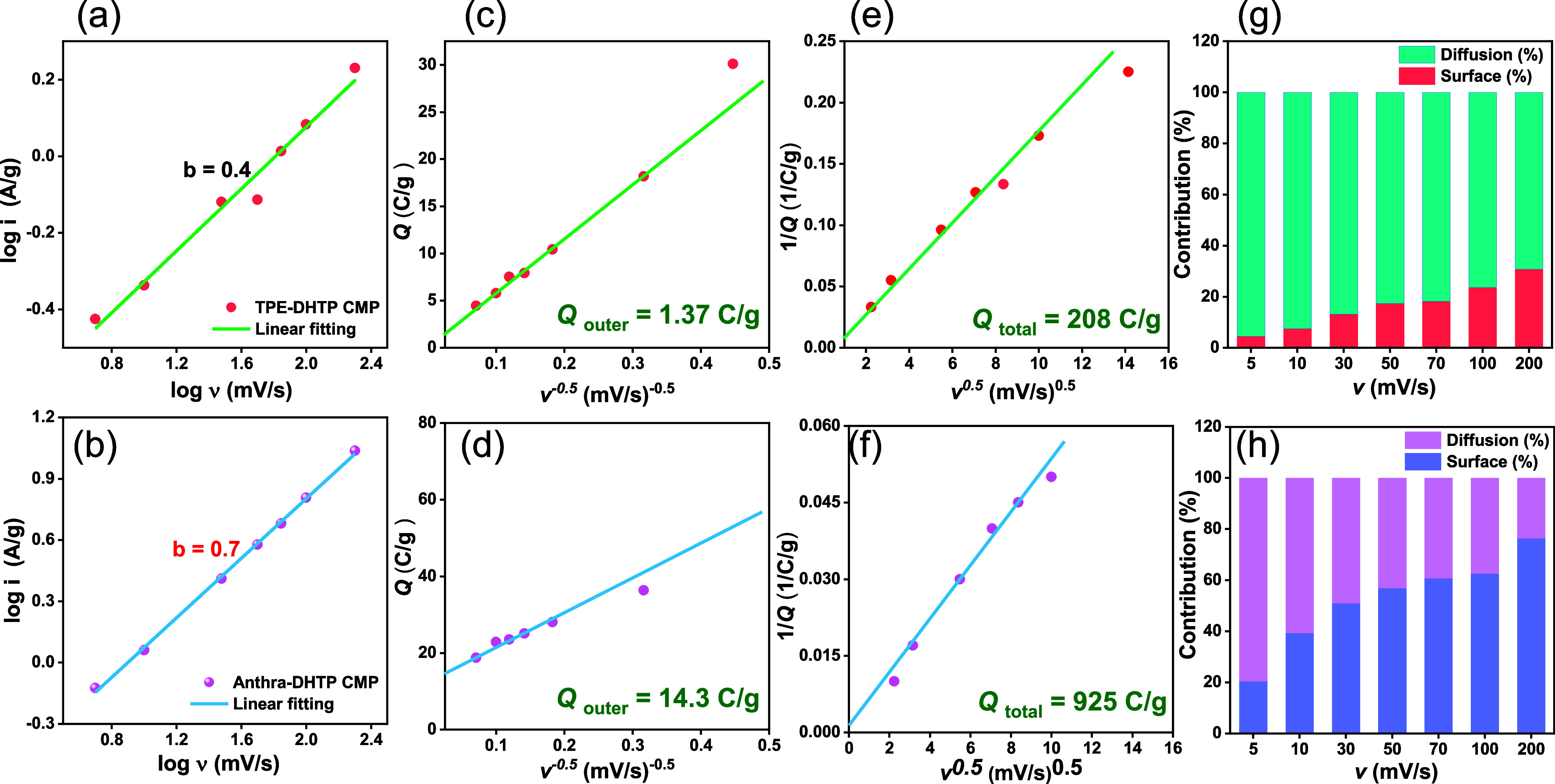
(a, b) Power’s law relation, (c, d) relation between *Q* (C g^–1^) and *v*^–0.5^ (mV s^–1^)^−0.5^, (e, f) 1/*Q* vs *v*^0.5^ (mV s^–1^)^−0.5^ for (a, c, e) TPE-DHTP and (b, d, f) Anthra-DHTP CMPs electrodes. Percentage of surface contribution and diffusion contribution for (g) TPE-DHTP and (h) Anthra-DHTP CMPs electrodes.

Using KOH (1 M) as the electrolyte in a three-electrode system, we initially investigated the electrochemical performance and mechanism of TPE-DHTP CMP and Anthra-DHTP CMP electrodes for supercapacitor applications. Our electrodes, made from these materials, were shaped like rectangles. The CV curves displayed humps over a potential window that varied with scan rate [Figure 6a,b]. As the potential scan rate increased, the peak current of both TPE-DHTP CMP and Anthra-DHTP CMP electrodes also rose. This indicated a small amount of charge transfer resistance, pseudocapacitance, and electric double-layer capacitance (EDLC) as the sources of their capacitive responses.^20,75^ The electron-rich phenyl rings and heteroatoms contributed to this characteristic, as evidenced by the humps

in the rectangular CV curves.^76^ The capacitive properties of Anthra-DHTP CMP are outstanding even at high scan rates, as presented by the CV curves for each electrode created at 50 mV s^–1^, shown in Figure 6c. The CV curve of Anthra-DHTP CMP, which has a larger integral area compared to that of TPE-DHTP CMP, indicates the best capacity for charge storage. Consequently, as illustrated in Figure 6d, Tables S1 and S2, the distinctive capacitance of Anthra-DHTP CMP is greater than that of TPE-DHTP CMP at various scan rates. The specific capacitance of Anthra-DHTP CMP is calculated to be 70, 36.34, 28, 25.1, 23.51, 22.83, and 18.8 C g^–1^ at 5, 10, 30, 50, 70, 100, and 200 mV s^–1^, respectively. Cyclic voltammetry (CV) is considered the most significant methodology for examining a material’s response to a voltage range, as it provides insight into its electrochemical behavior, voltage procedure, reversible nature (diffusion vs surface control), and energy storage mechanism. After reviewing the electrochemical findings, the capacitive aspect of total charge storage was investigated. The power law was utilized to analyze the electrodes’ charge storage capability. This analysis can be expressed either as log *i* = *a* + *b* log *v* or *i*(*V*) = *av^b^*, where *i* represents the applied current density and v the applied potential window. The intercept and slope of the log *i* vs log *v* plots are used to determine the parameters *a* and *b*, which are constants. The value of *b* is obtained from the slope of the linear fit of the log *i* vs log *v* plot at a fixed voltage. Figure 7a,b depict the plot of log *i* versus log *v*, showing that the *b* value (∼0.4) is closer to 0.5. This indicates that ion intercalation is the dominant mechanism for charge storage. Conversely, a *b* value higher than 0.5 suggests that under these conditions, the capacitive contribution is more prevalent than the intercalation process. Additionally, the kinetics of charge storage in TPE-DHTP CMP and Anthra-DHTP CMP have been further examined using the Trasatti technique. Two different charge storage processes are found in this analysis: the buildup of charge on the outside surface of particles (capacitive) is frequently referred to as *Q*_outer_ (outer surface) and the first is named *Q*_bulk_ (inner surface).
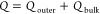


Figure 7c,d show the relation among capacity *Q* and (*v*)^−0.5^, where (*v*) is the possible scan rate. By finding an intercept of the graph of *Q* and (*v*)^−0.5^, it can be done to determine the value of (*Q*_outer_) by the equation:
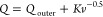
where *K* is a constant, *v* (mV s^–1^) is the potential scan rate, and *Q* (C g^–1^) is the capacity produced by every single CV sequence. It is determined that the maximum sweep-rate capacitance of TPE-DHTP CMP and Anthra-DHTP CMP is 1.37 and 14.3 C g^–1^.

This figure represents the stored charge because of double-layer and/or pseudocapacitance capacitive mechanisms. This process takes place when the potential scan rate gets to the lowest possible levels, giving the ions sufficient time to disperse. Plotting 1/*Q* against (*v*)^0.5^ yields the total charge (*Q*_total_), as seen in Figure 7e,f. Based on the relationship:
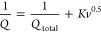
However, in this case, we can calculate the stored charge which is 208 and 952 C g^–1^ for TPE-DHTP CMP and Anthra-DHTP CMP; respectively. The charge stored increases based on previously published techniques when the surface contribution rises, and diffusion limitation takes precedence over the scan rate. The percentage of surface and diffusion-controlled contributions at scan rates are thus displayed in Figure 7g,h.

**Figure 8:**
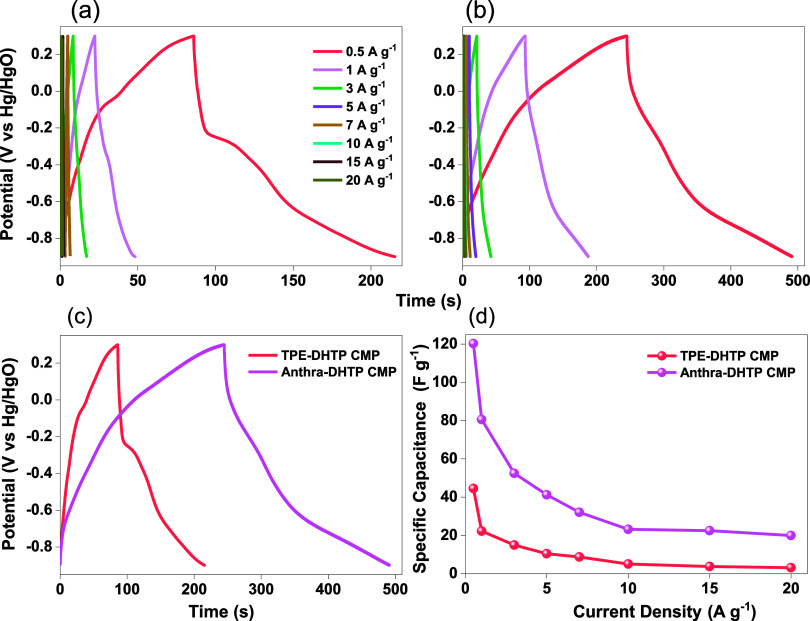
GCD curves of (a) TPE-DHTP and (b) Anthra-DHTP CMPs, (c) CGD curve of DHTP-CMPs electrodes were obtained using 0.5 A g^–1^ and (d) specific capacitances for DHTP-CMPs electrodes were calculated from GCD.

Diffusion-controlled faradic yields are 95.4 and 79.5% for TPE-DHTP CMP and Anthra-DHTP CMP electrodes. In Figure 8a,b, TPE-DHTP CMP and Anthra-DHTP CMP electrodes underwent galvanostatic charge–discharge (GCD) tests. The resulting GCD curves, which were modestly bent and triangular, suggested characteristics of both pseudocapacitance and EDLC, consistent with the CV curve data. The noticeable potential drop at the onset of each discharge curve in the GCD profiles for the TPE-DHTP CMP and Anthra-DHTP CMP electrodes (Figure 8a,b) can be attributed to the internal resistance within the electrode material.^76,77^Figure 8c compares the GCD curves of DHTP-CMPs at 0.5 A g^–1^. It is observed that the Anthra-DHTP CMP materials exhibited longer discharge durations than the TPE-DHTP CMP materials. This suggests excellent capacitive behavior and good reversibility of charge/discharge cycles, indicating that the capacitance of the Anthra-DHTP CMP material was significantly enhanced. We determined the capacitances of the TPE-DHTP and Anthra-DHTP CMPs samples from their GCD profiles. 0.5 A g^–1^, the specific capacitance of Anthra-DHTP CMP is 121 F g^–1^, while that of TPE-DHTP CMP is 44 F g^–1^ (Figure 8c). According to the GCD data (Figure 8d), the specific capacitances of Anthra-DHTP CMP were 121, 81, 62, 53, 41, 32, 23, 23, and 20 F g^–1^, respectively [measured at 0.5, 1, 2, 3, 5, 7, 10, 15, and 20 A g^–1^] are which aligns with the outcomes of the CV tests.

**Figure 9:**
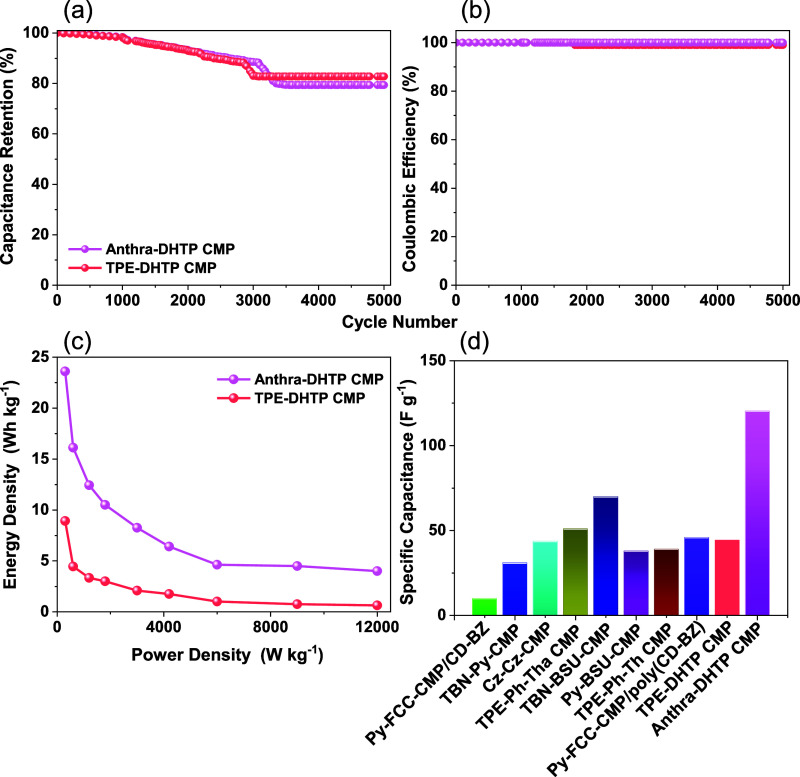
(a) The stability test, (b) Coulombic efficiency, (c) Ragone plot for Anthra-DHTP and TPE-DHTP CMPs, and (d) specific capacitances of the Anthra-DHTP and TPE-DHTP CMPs and those of other CMP materials previously reported for supercapacitors.

Continuous cycling experiments are valuable for assessing the longevity of electrode materials. After undergoing 5000 consecutive charge–discharge cycles, the capacitance stability (*C*_s_) of Anthra-DHTP CMP and TPE-DHTP CMP samples maintained 79 and 82% of their original capacitance and their Coulombic efficiency remained close to 100%, as shown in Figure 9a,b. The existence of heteroatoms and increased surface area are responsible for these results, which enhance the electrode–electrolyte interaction, and facilitate the flow of electrolyte ions.

**Figure 10:**
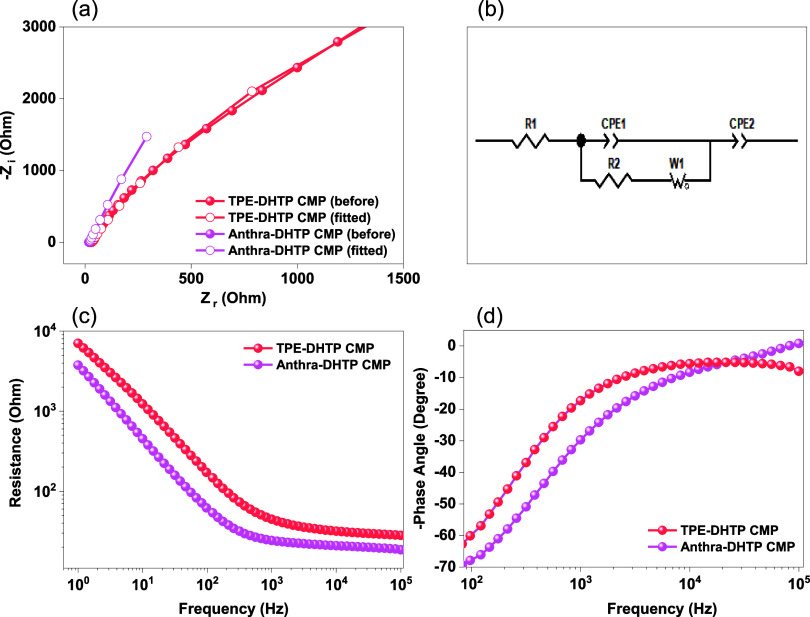
EIS curves: (a) Nyquist plots, (b) equivalent fitted circuit, (c) Bode plot of frequency-dependent resistance (magnitude), and (d) Bode plot of frequency-dependent phase angles of TPE-DHTP and Anthra-DHTP CMPs.

Furthermore, the energy densities of the materials might be determined using the Ragone plot (Figure 9c). At specific power (SP) of 300 W kg^–1^, for both DHTP-CMPs materials, indicating energy densities of 8.9 and 23.6 Wh kg^–1^ for TPE-DHTP and Anthra-DHTP CMPs. Anthra-DHTP CMP exhibits a maximum energy density of 23.6 Wh kg^–1^, attributed to its enhanced surface area and porosity. These results are promising and show the material’s potential for SC applications. Table S3 presents an examination of DHTP CMPs electrodes, as well as other porous materials that have been reported to be employed in supercapacitor applications (Figure 9d). In this study, we utilized KOH electrolyte (1 M) to conduct Electrochemical Impedance Spectroscopy (EIS) measurements on our DHTP-CMPs. Table S4 presents the fitted data, which allowed us to generate Nyquist plots and corresponding electrical equivalent circuits for Anthra-DHTP CMP and TPE-DHTP CMP, depicted in Figure 10a,b. The EIS data was fitted to circuit models that included parameters such as *Z*_W_ (Warburg element), *R*_ct_ (charge transfer resistance), *R*_s_ (series resistance), and two constant phase elements (CPE-EDL, CPE-P). The initial *R*_s_ values for TPE-DHTP CMP and Anthra-DHTP electrodes were recorded at 30.7 and 20.9 Ω, respectively [Table S4]. These relatively low ohmic resistance values indicate their superior capacitance. Additionally, Figure 10c illustrates the frequency-dependent magnitude Bode plot, highlighting the exceptional capacitive behavior of these DHTP-CMPs and emphasizing their potential in energy applications. Furthermore, Figure 10d displays the frequency-dependent phase angle Bode plot, revealing knee frequencies that Act as metrics for evaluating the performance rate of electrode materials. The moderate knee frequencies observed for TPE-DHTP CMP and Anthra-DHTP CMP suggest that these DHTP-CMPs could be effective electrodes for various energy-related applications, demonstrating both capacitive and resistive characteristics. Figure S13 displays the results of EIS analysis for the DHTP-CMPs after 5000 cycles, presented through Nyquist plots across a frequency range from 100 kHz to 10 mHz. As observed, there was an observed increase in ion diffusion resistance, indicating a slowdown in ion diffusion and the slope of the straight line in the low-frequency region decreased (showing a less vertical line), suggesting an increase in ion diffusion resistance. Additionally, after 5000 cycles, the semicircles in the high-frequency region (indicating charge transfer resistance) became more pronounced. These observations imply a potential loss of contact between the substrate and active material over multiple cycles. Based on these findings, Anthra-DHTP CMP demonstrated superior cycling performance and reversibility compared to TPE-DHTP CMP.

## Conclusions

In conclusion, we successfully synthesized two distinct forms of DHTP-CMPs featuring CH=N and phenolic units, TPE-DHTP CMP and Anthra-DHTP CMP, using the efficient and simple Schiff-base condensation reaction. Both DHTP-CMPs exhibit good thermal stability and porous properties, with Anthra-DHTP CMP standing out for its exceptional char yield: 68 wt % and *T*_d10_ = 505 °C. Compared to TPE-DHTP CMP, Anthra-DHTP CMP possesses the largest BET_SA_ of 431 m^2^ g^–1^ and the highest CO_2_ absorption capacity at 273 K of 1.85 mmol g^–1^ (8.14 wt %). In electrochemical testing, TPE-DHTP CMP and Anthra-DHTP CMP showed capacitance values of 44 and 121 F g^–1^, respectively, at 0.5 A g^–1^, attributed to the behavior of their heteroatoms and excellent porosity. Therefore, these results confirm that Anthra-DHTP CMP is an ideal material choice for energy storage and gas capture applications. This work demonstrates that the efficient condensation process used to produce DHTP-CMPs has applications extending beyond CO_2_ absorption and supercapacitors. Additionally, these materials show potential for use in photocatalysis, iodine and dye capture, and other related fields.

## Supporting Information Available

Additional experimental details, characterizations, electrochemical analysis, measurements, and schematic diagrams of TPE-4NH_2_, Anthra-Br_4_, and Anthra-4Ph-4NH_2_, along with FTIR and NMR data for TPE-4NH_2_, Anthra-Br_4_, and Anthra-4Ph-4NH_2_; data on solubility, XPS, XRD, and EIS for TPE-DHTP CMP and Anthra-DHTP CMP; as well as a performance comparison of supercapacitors using TPE-DHTP CMP and Anthra-DHTP CMP electrodes with previously described electrodes (PDF)
