# Supplementary material for: Tetraphenylanthraquinone and Dihydroxybenzene-Tethered Conjugated Microporous Polymer for Enhanced CO2 Uptake and Supercapacitive Energy Storage
Source: JACS Au. 2024 Aug 16;4(9):3593–605. doi: 10.1021/jacsau.4c00537 (PMC11423306; doi:10.1021/jacsau.4c00537)
Supplement: Supplementary file 1 — au4c00537_si_001.pdf [file au4c00537_si_001.pdf]

Supporting Information for

Tetraphenylanthraquinone and Dihydroxybenzene-Tethered Conjugated Microporous Polymer for Enhanced CO_2 Uptake and Supercapacitive Energy Storage

Mohamed Gamal Mohamed[a,b], Chia-Chi Chen[a], Mervat Ibrahim[c], Aya Osama Mousa[a], Mohamed Hammad Elsayed[d], Yunsheng Ye[a], and Shiao-Wei Kuo[a,e]*

[a]Department of Materials and Optoelectronic Science, College of Semiconductor and Advanced Technology Research, Center for Functional Polymers and Supramolecular Materials, National Sun Yat-Sen University, Kaohsiung 804, Taiwan.

[b]Department of Chemistry, Faculty of Science, Assiut University, Assiut 71516, Egypt.

[c]Chemistry Department, Faculty of Science, New Valley University, El-Kharja, 72511, Egypt.

[d]Department of Chemistry, Faculty of Science, Al-Azhar University, Nasr City, Cairo 11884, Egypt

[e]Department of Medicinal and Applied Chemistry, Kaohsiung Medical University, Kaohsiung 807, Taiwan.

Corresponding authors:

E-mail: kuosw@faculty.nsysu.edu.tw (S. W. Kuo).

Characterization

FTIR spectra were collected on a Bruker Tensor 27 FTIR spectrophotometer with a resolution of 4 cm^{-1} by using the KBr disk method. ^{13}C nuclear magnetic resonance (NMR) spectra were examined by using an INOVA 500 instrument with DMSO-d_6 and $CDCl_3$ as the solvent and TMS as the external standard. Chemical shifts are reported in parts per million (ppm). Solid-state ^{13}C NMR was measured by JEOL JNM-LA300 spectrometer and standard CPMAS probe at 75.577 MHz. The thermal stabilities of the samples were performed by using a TG Q-50 thermogravimetric analyzer under a N_2 atmosphere; the sample (ca. 5 mg) was put in a Pt cell with a heating rate of 20 °C min^{-1} from 100 to 800 °C under a N_2 flow rate of 60 mL min^{-1}. Solid-state ^{13}C NMR was measured by JEOL JNM-LA300 spectrometer and standard CPMAS probe at 75.577 MHz. The morphologies of the polymer network samples were examined by Field emission scanning electron microscopy (FE-SEM; JEOL JSM7610F) and transmission electron microscope (TEM) using a JEOL-2100 instrument at an accelerating voltage of 200 kV. X-ray Photoelectron Spectroscopy (XPS): XPS was measured on a X-ray Photoelectron Spectrometer System (Thermo Scientific). The X-ray monochromator used micro-focused Al-Kα radiation. Surface area and porosity measurements of samples weighing approximately 40-60 mg were conducted using the BEL MasterTM/BEL simTM (version 3.0.0) apparatus. Nitrogen (N_2) adsorption and desorption isotherms were generated by gradually exposing the samples to ultrahigh-purity N_2 gas, reaching pressures of up to about 1 atmosphere, while maintaining a temperature of 77 K in a liquid nitrogen bath. Before these measurements, the samples underwent a degassing process at 150 °C for 8 h. The instrument's software was utilized to calculate surface parameters using the BET adsorption models. Furthermore, the pore size of the prepared samples was determined using nonlocal density functional theory (NLDFT).

Electrochemical Analysis

Working Electrode Cleaning: Before use, the glassy carbon electrode (GCE) was polished several times with 0.05-μm alumina powder, washed with EtOH after each polishing step, cleaned through sonication (5 min) in a water bath, washed with EtOH, and then dried in air.

Electrochemical Characterization: The electrochemical experiments were performed in a three-electrode cell using an Autolab potentiostat (PGSTAT204) and 1 M KOH as the aqueous electrolyte. The GCE was used as the working electrode (diameter: 5.61 mm; 0.2475 cm^2); a Pt wire was used as the counter electrode; Hg/HgO (RE-1B, BAS) was the reference electrode. All reported potentials refer to the Hg/HgO potential. A slurry was prepared by dispersing the TPE-DHTP CMP or Anthra-DHTP CMP (50%), carbon black (40%), and Nafion (10%) in a mixture of (EtOH/ H_2O) (200 μL: 800 μL) and then sonicated for 2 h. A portion of this slurry (10 μL) was pipetted onto the tip of the electrode, which was then dried in air for 30 min before use. The electrochemical performance was studied through CV at various sweep rates (5–200 mV s^{-1}) and through the GCD method in the potential range of +0 to -1 V at varying current densities (0.5-20 A/g) and CV at different sweep rates (5-200 mV/sec). The equation below was used to compute the specific capacitance based on the CV curves:

$$Q = \frac{\int \mathrm{IdV}}{2\ m\, v}$$

The specific capacitance was calculated from the GCD data using the equation:

$$C_s = (I\Delta t)/(m\Delta V)$$

Where C_s (F g^{-1}) is the specific capacitance of the supercapacitor, I (A) is the discharge current, ΔV (V) is the potential window, Δt (s) is the discharge time, and m (g) is the mass of the NPC on the electrode.

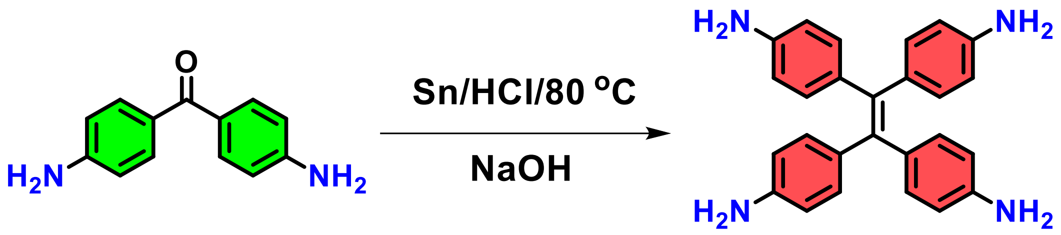

Scheme S1. Synthesis of TPE-4NH_2.

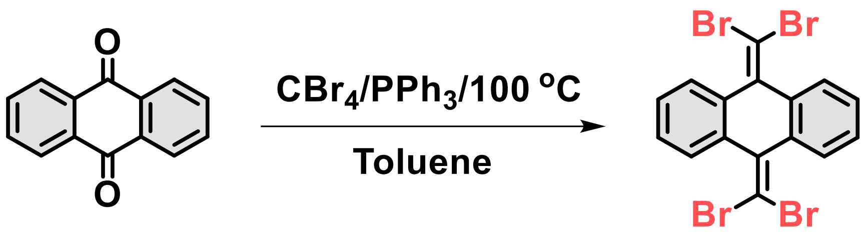

Scheme S2. Synthesis of Anthra-Br_4.

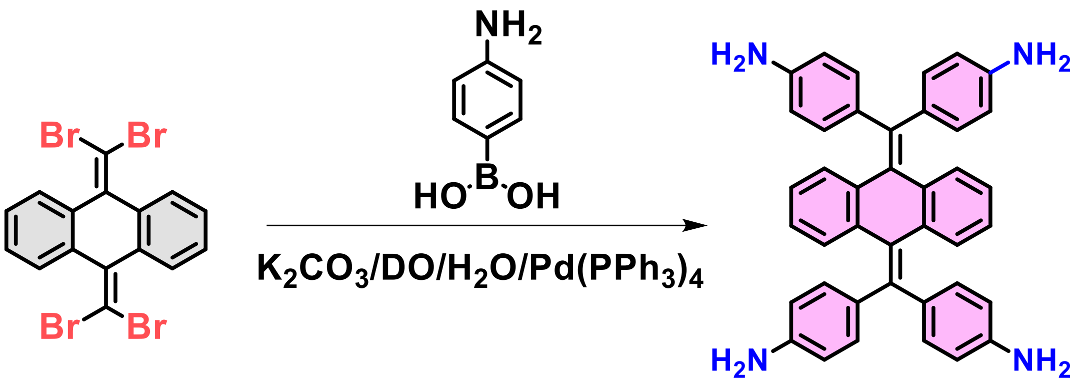

Scheme S3. Synthesis of Anthra-4Ph-4NH_2.

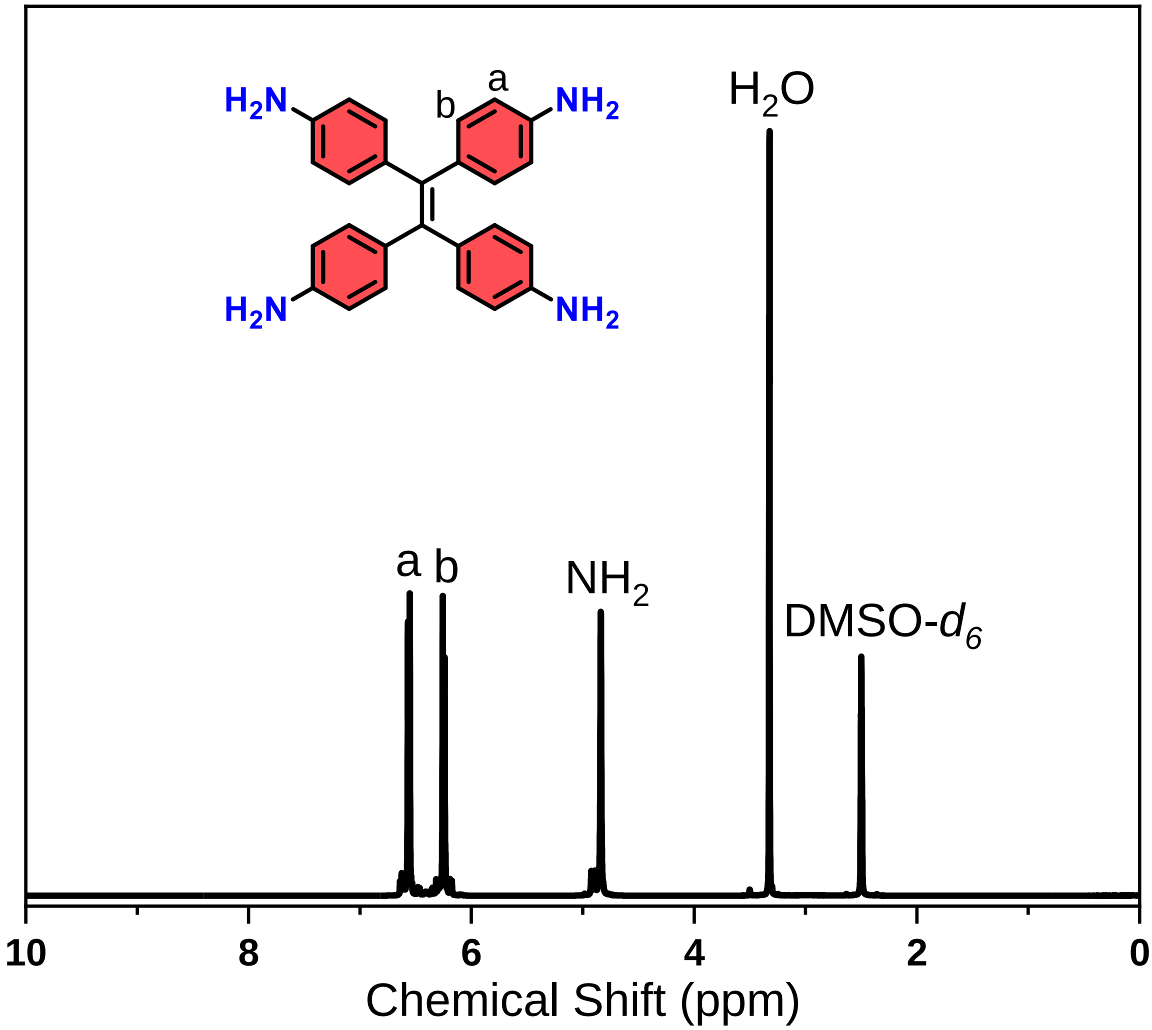

Figure S1. ^{1}H NMR spectrum of TPE-4NH_2.

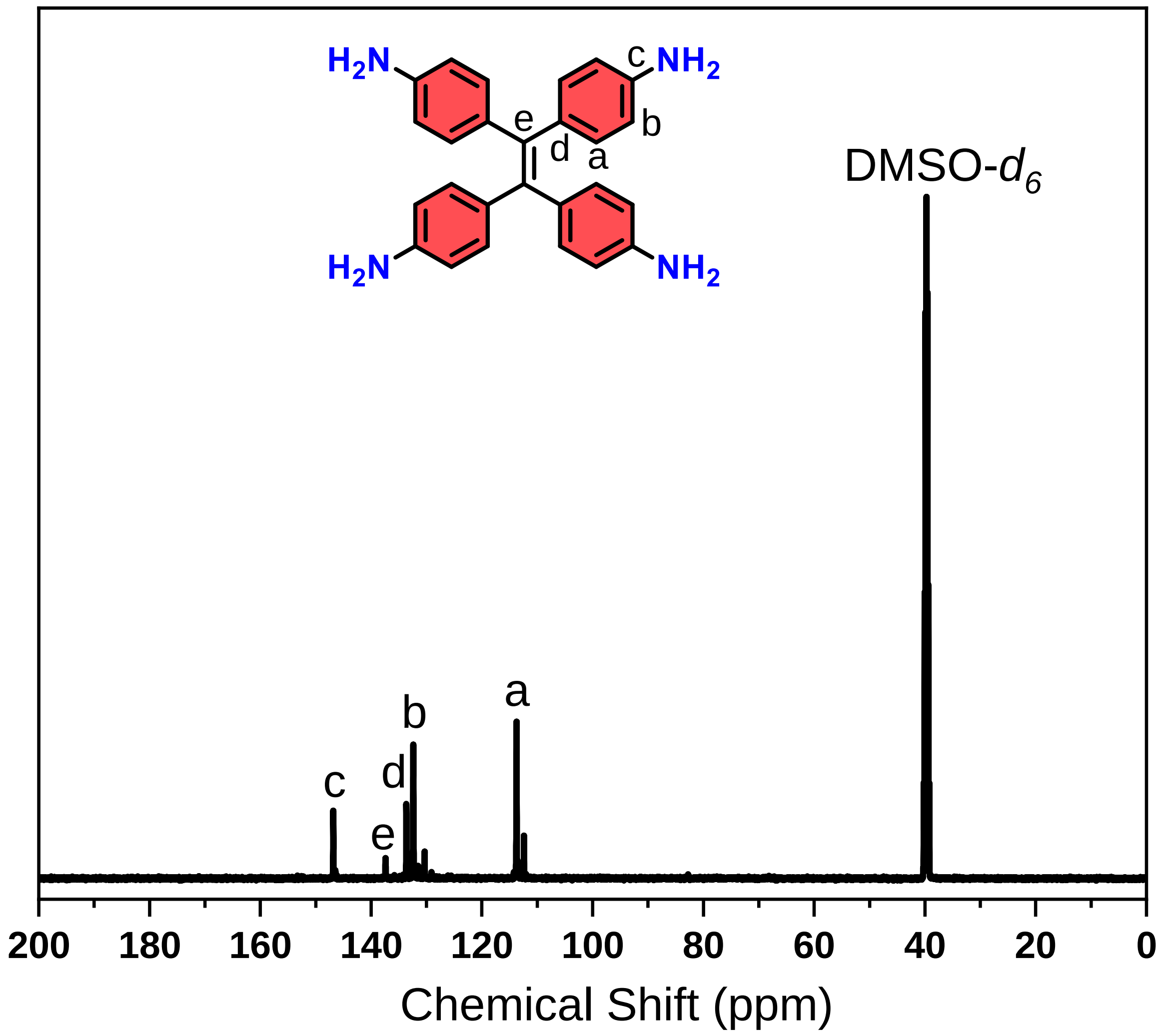

Figure S2. ^{13}C NMR spectrum of TPE-$4NH_2$.

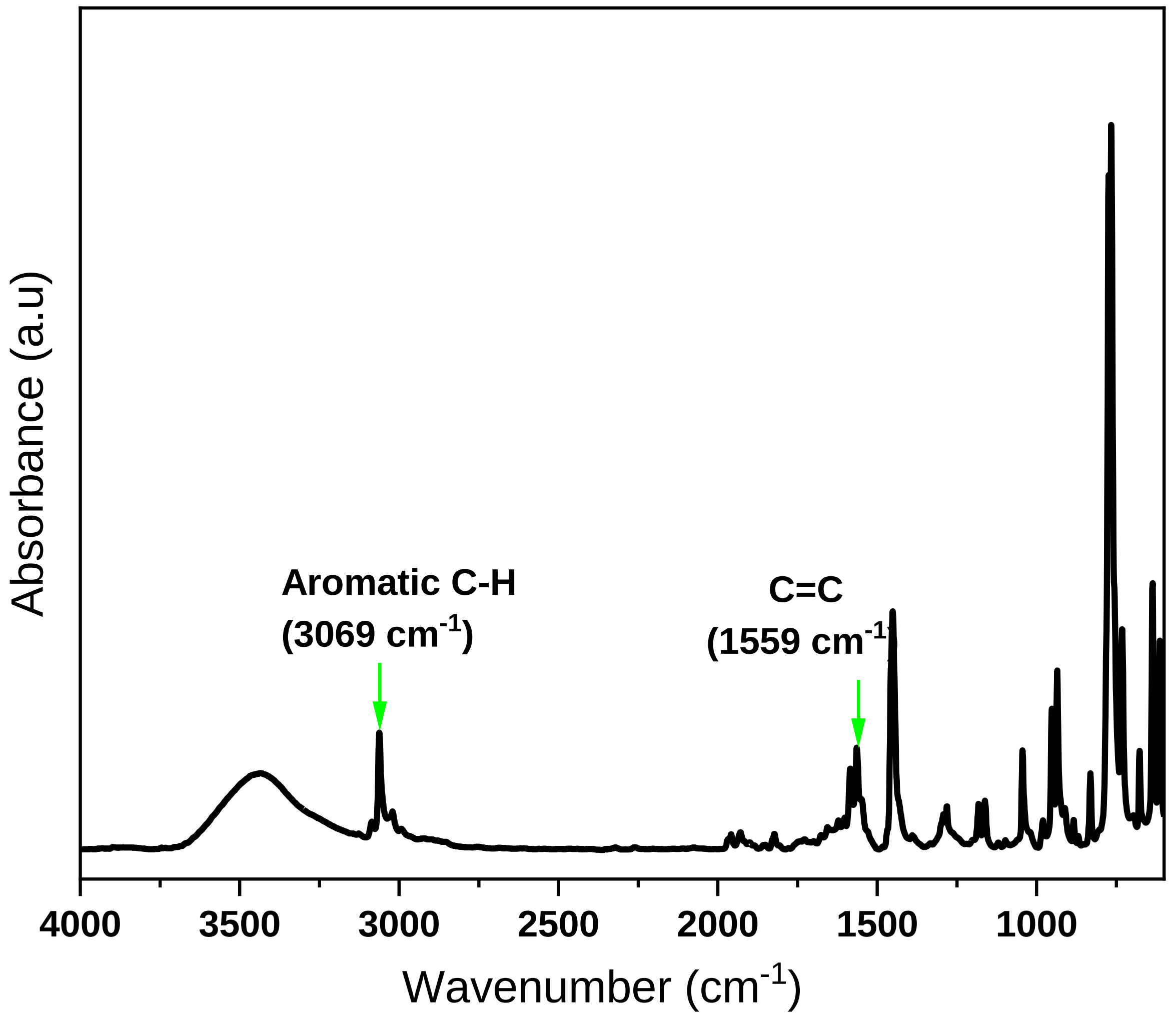

Figure S3. FTIR spectrum of Anthra-Br_4.

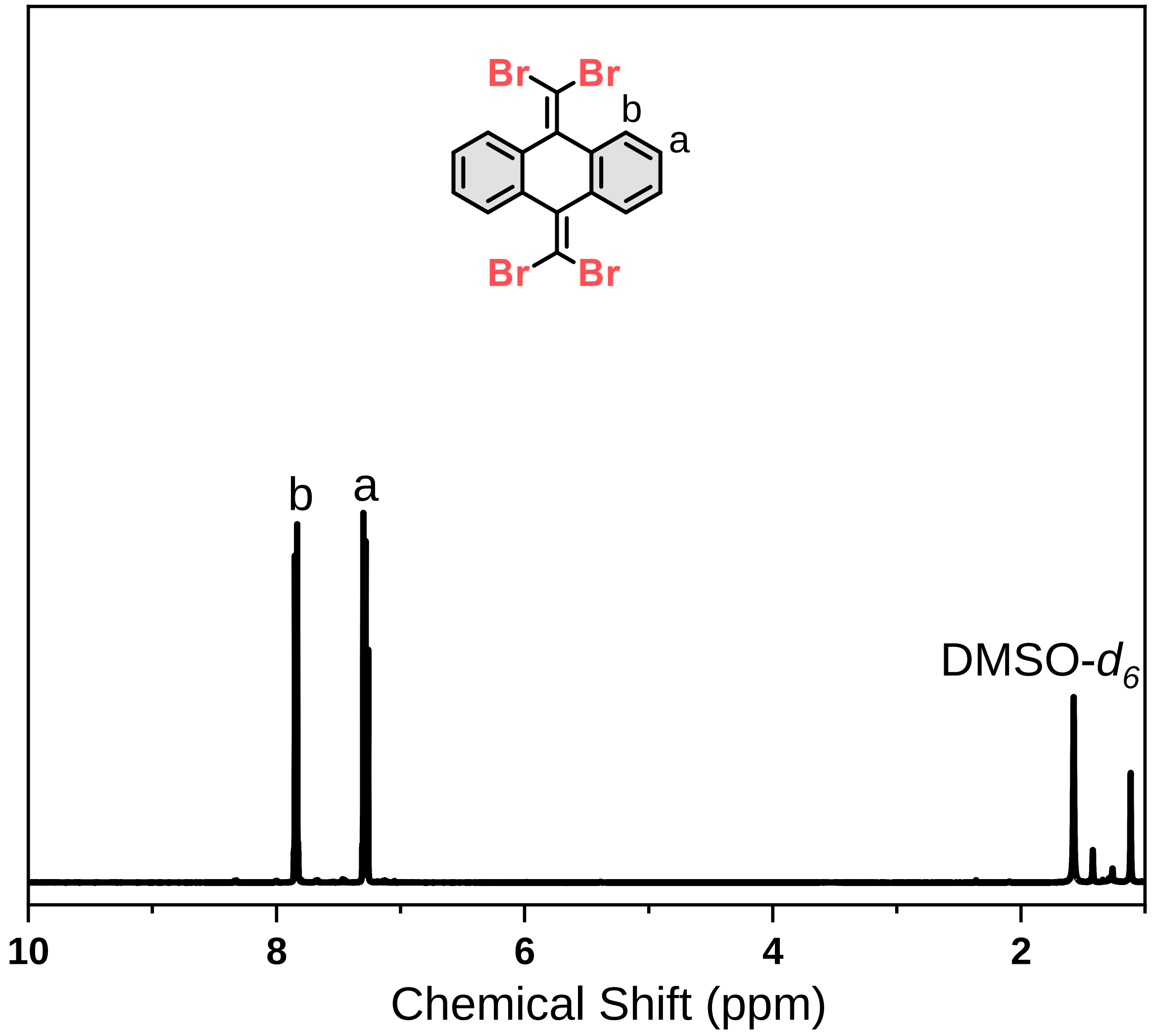

Figure S4. ^{1}H NMR spectrum of Anthra-Br_4.

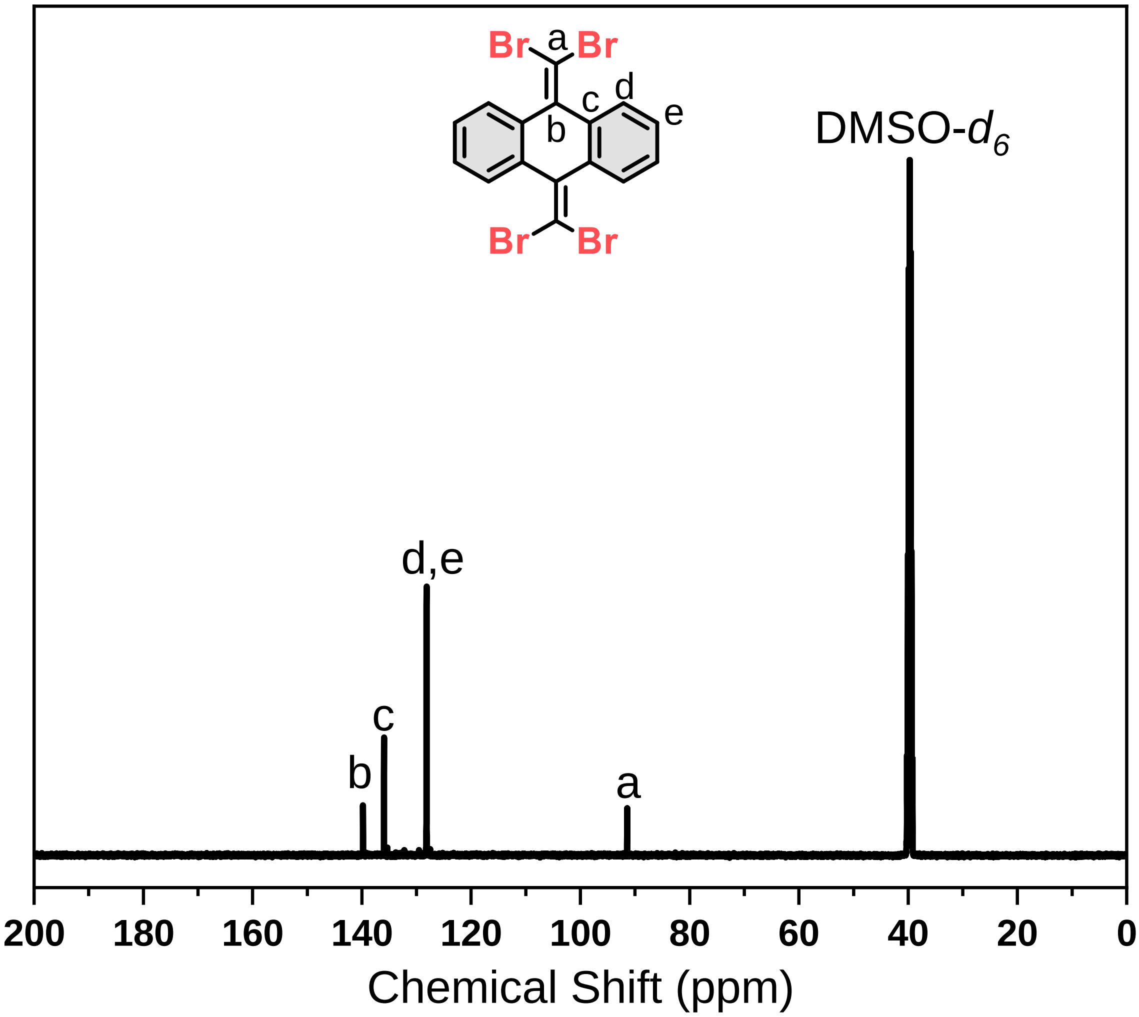

Figure S5. ^{13}C NMR spectrum of Anthra-Br_4.

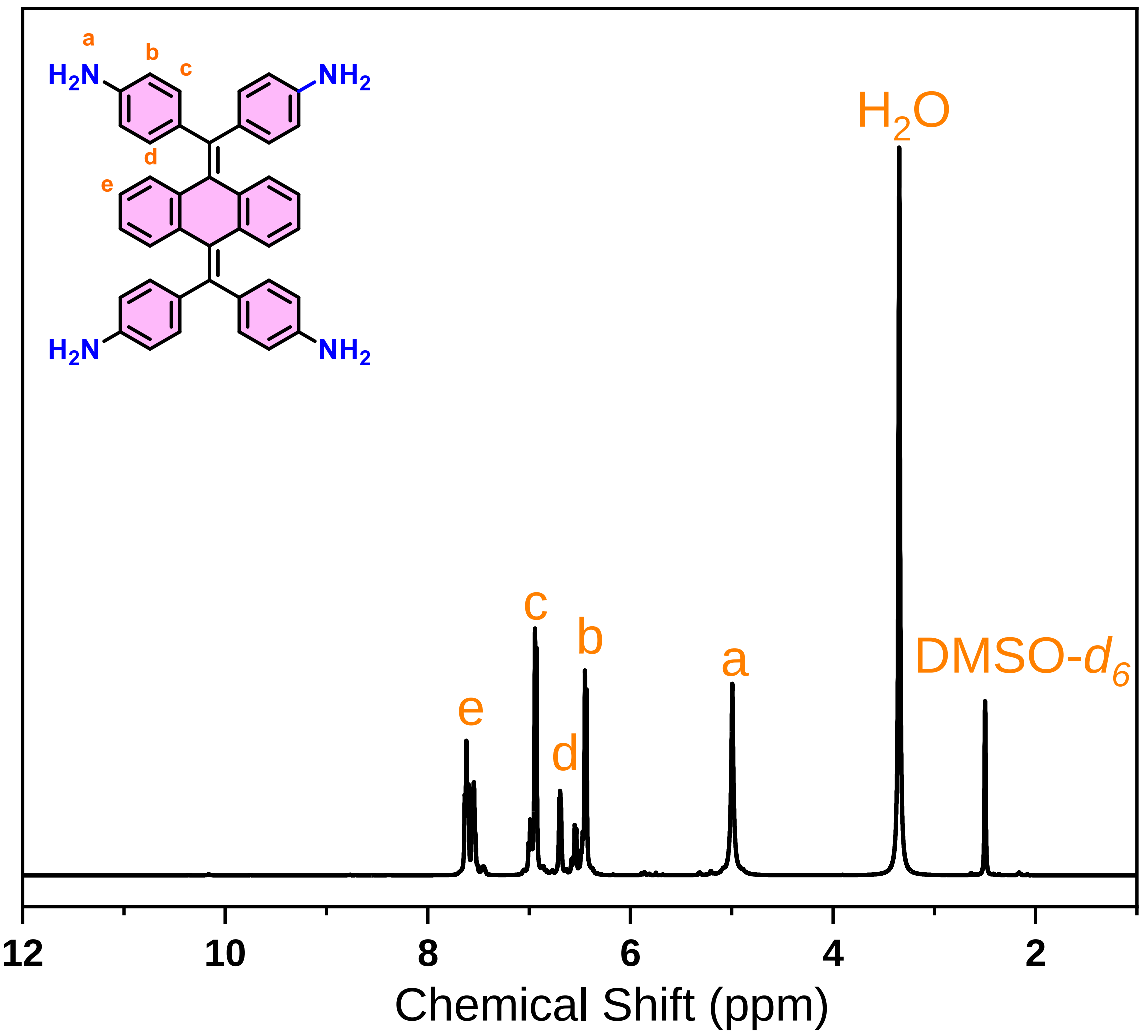

Figure S6. ^{1}H NMR spectrum of Anthra-4Ph-4NH_2.

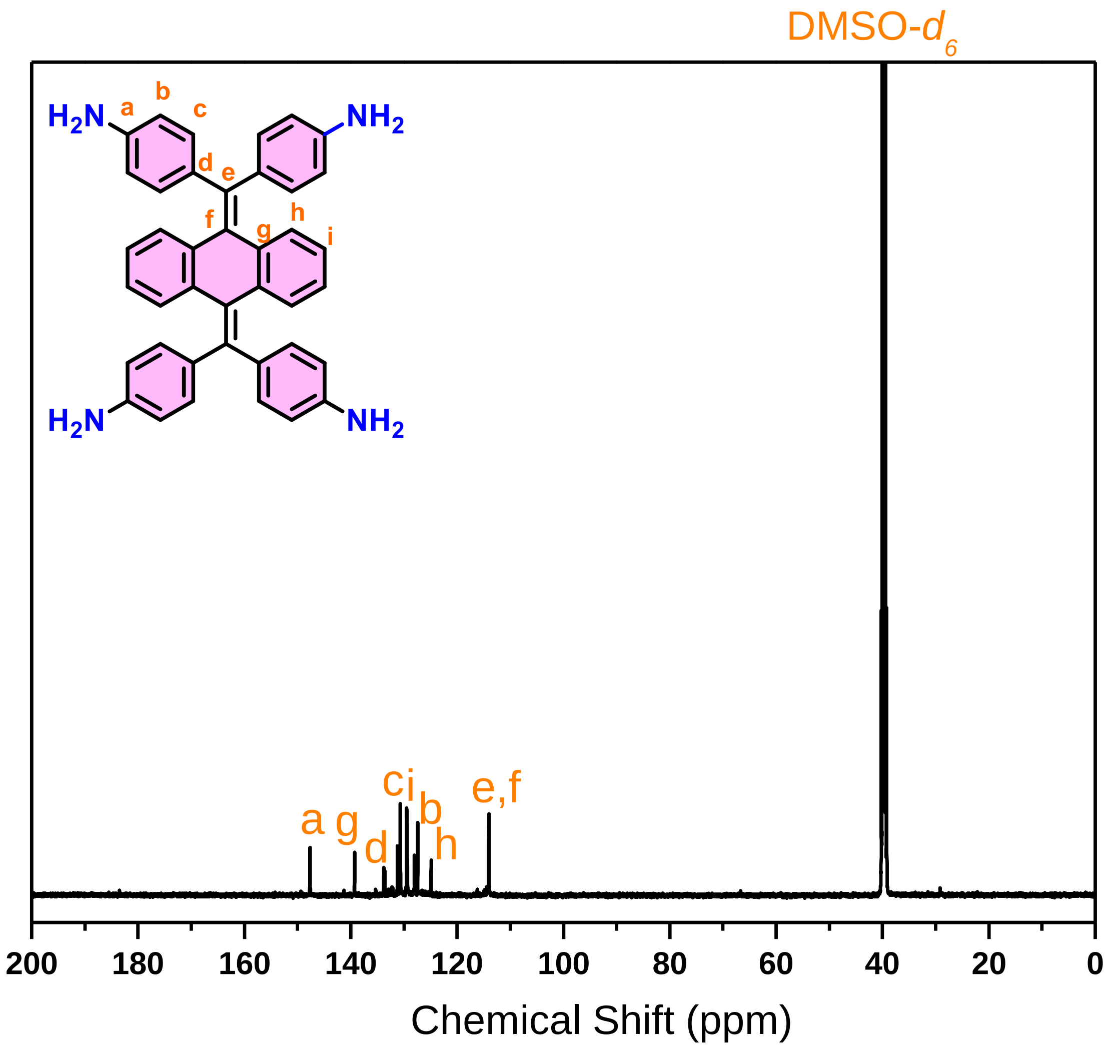

Figure S7. ^{13}C NMR spectrum of Anthra-4Ph-4NH$_2$.

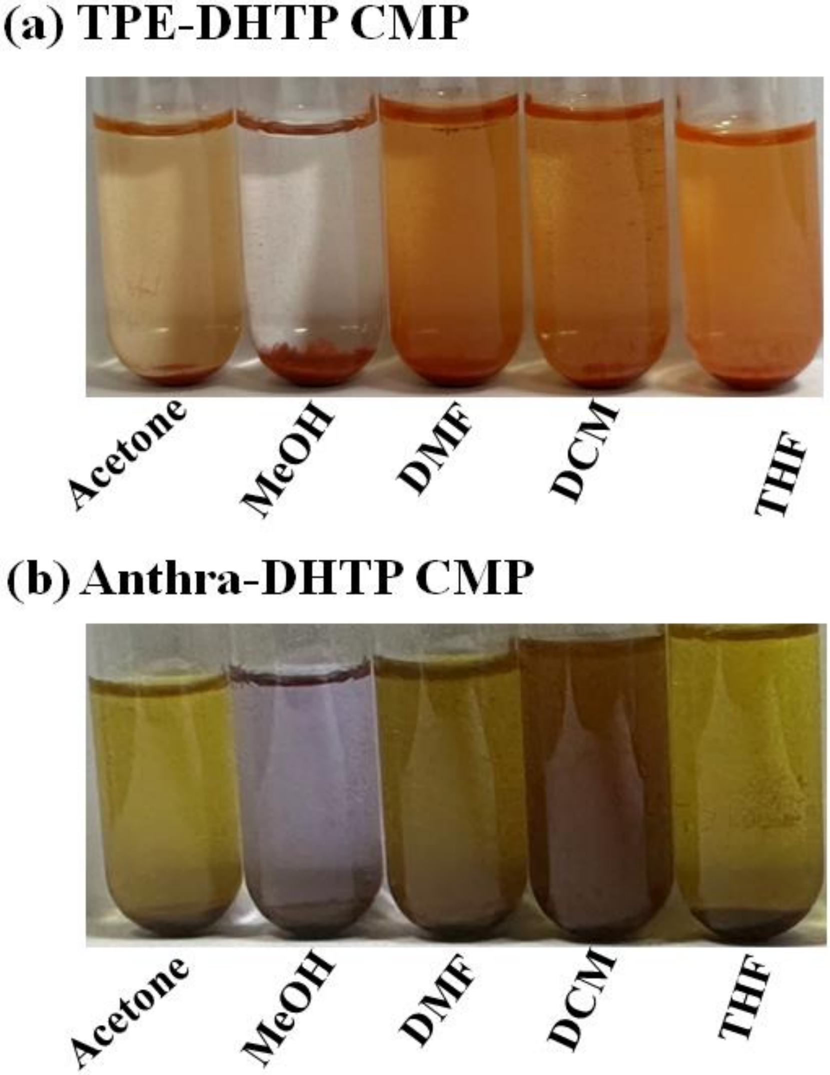

Figure S8. The solubility of (a) TPE-DHTP and (b) Anthra-DHTP CMPs was tested in various organic solvents including acetone, MeOH, DMF, DCM, and THF.

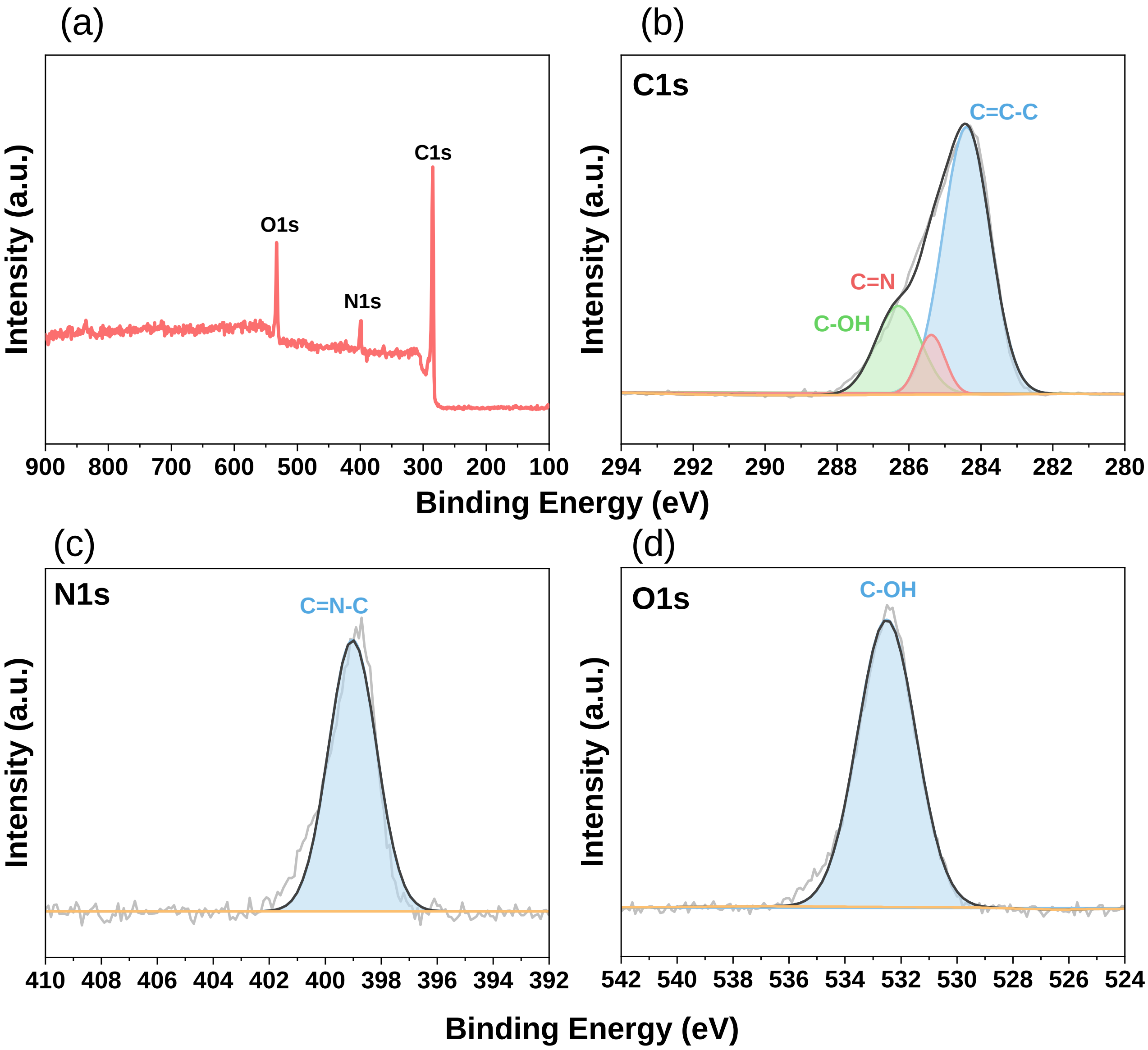

Figure S9. (a) High-resolution XPS spectra of TPE-DHTP CMP. XPS fitting data of (b) C1s, (c) N 1s, and (d) O1s spectra of TPE-DHTP CMP.

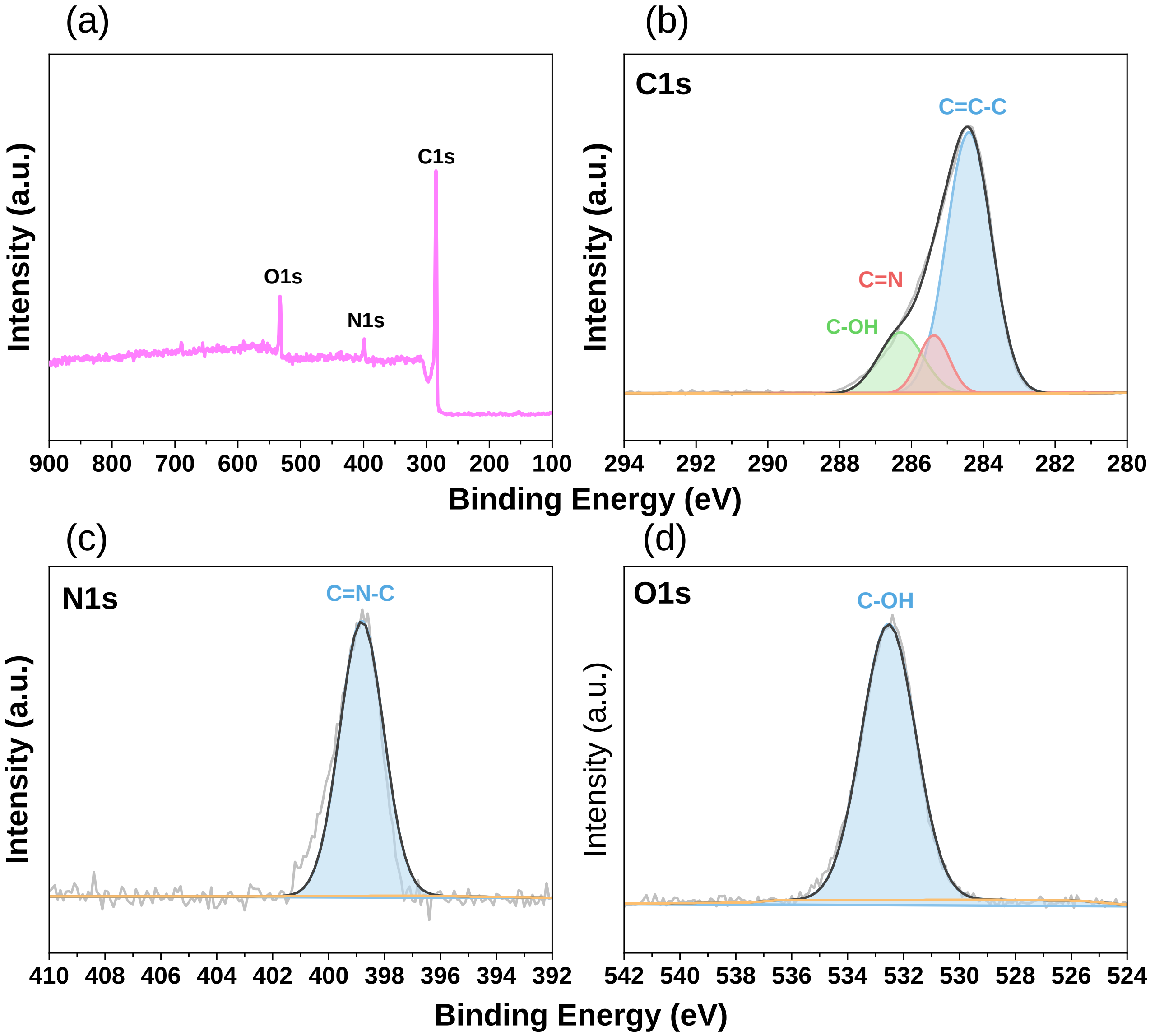

Figure S10. (a) High-resolution XPS spectra of Anthra-DHTP CMP. XPS fitting data of (b) C1s, (c) N 1s, and (d) O1s spectra of Anthra-DHTP CMP.

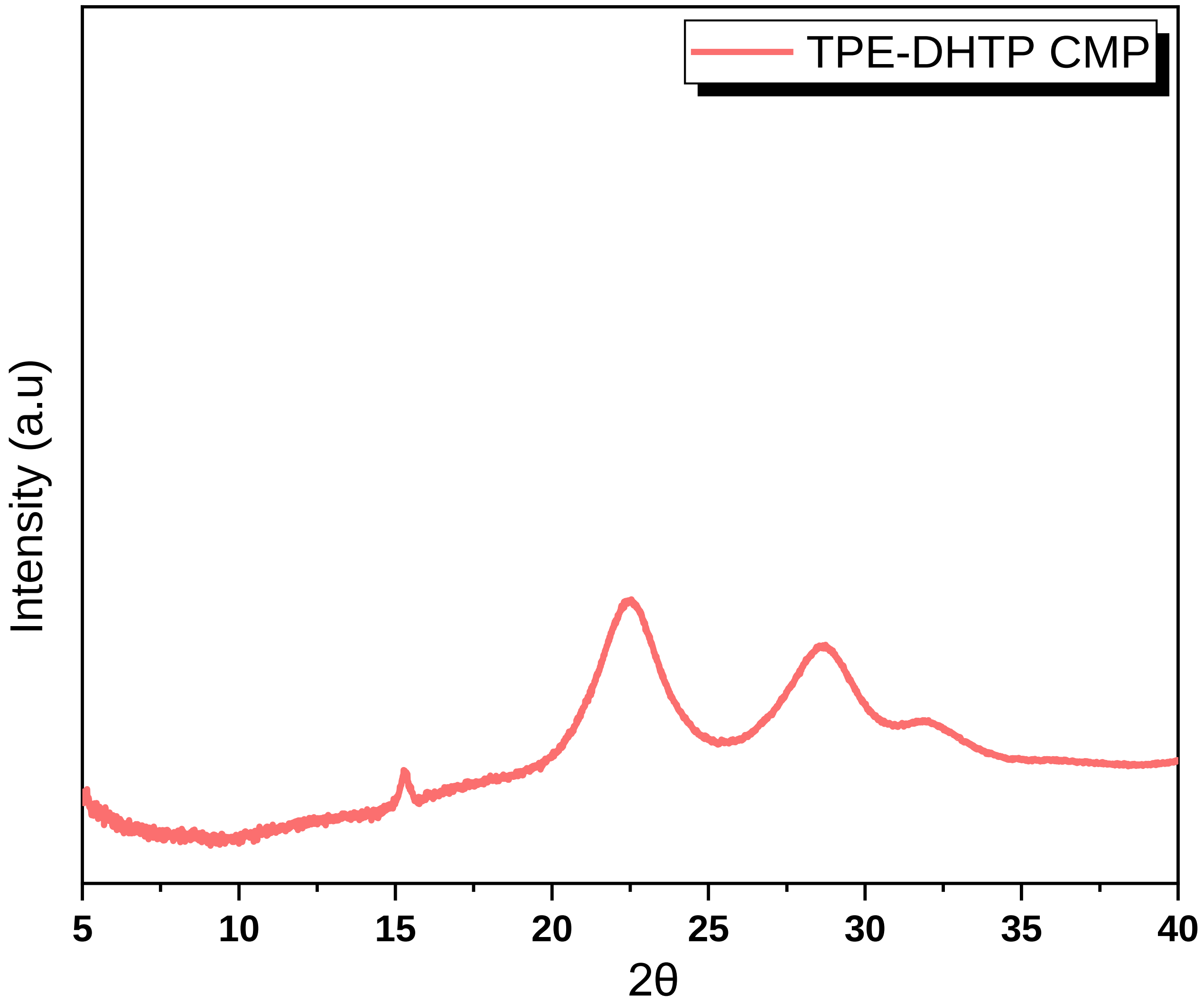

Figure S11. XRD spectrum of TPE-DHTP CMP.

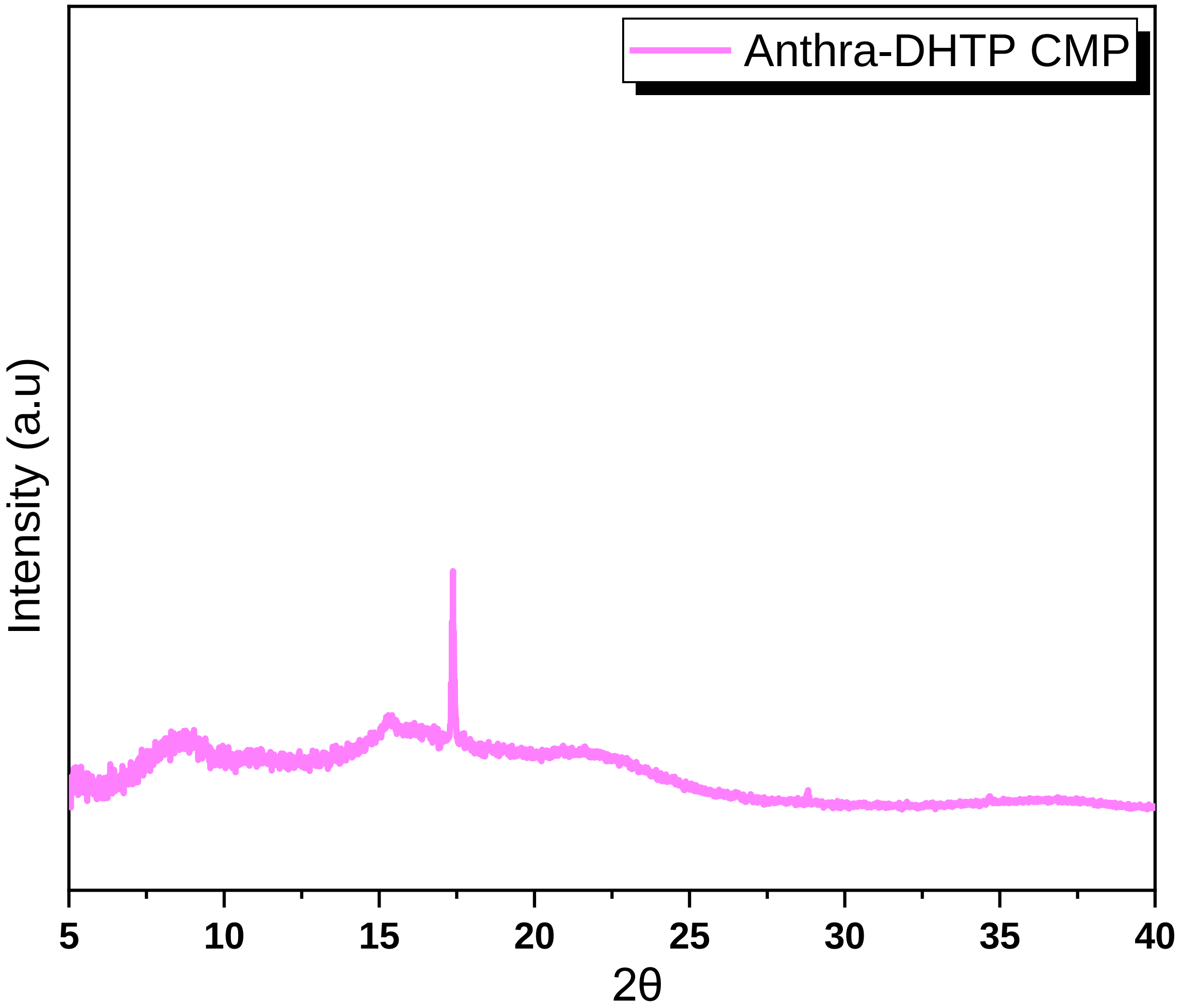

Figure S12. XRD spectrum of Anthra-DHTP CMP.

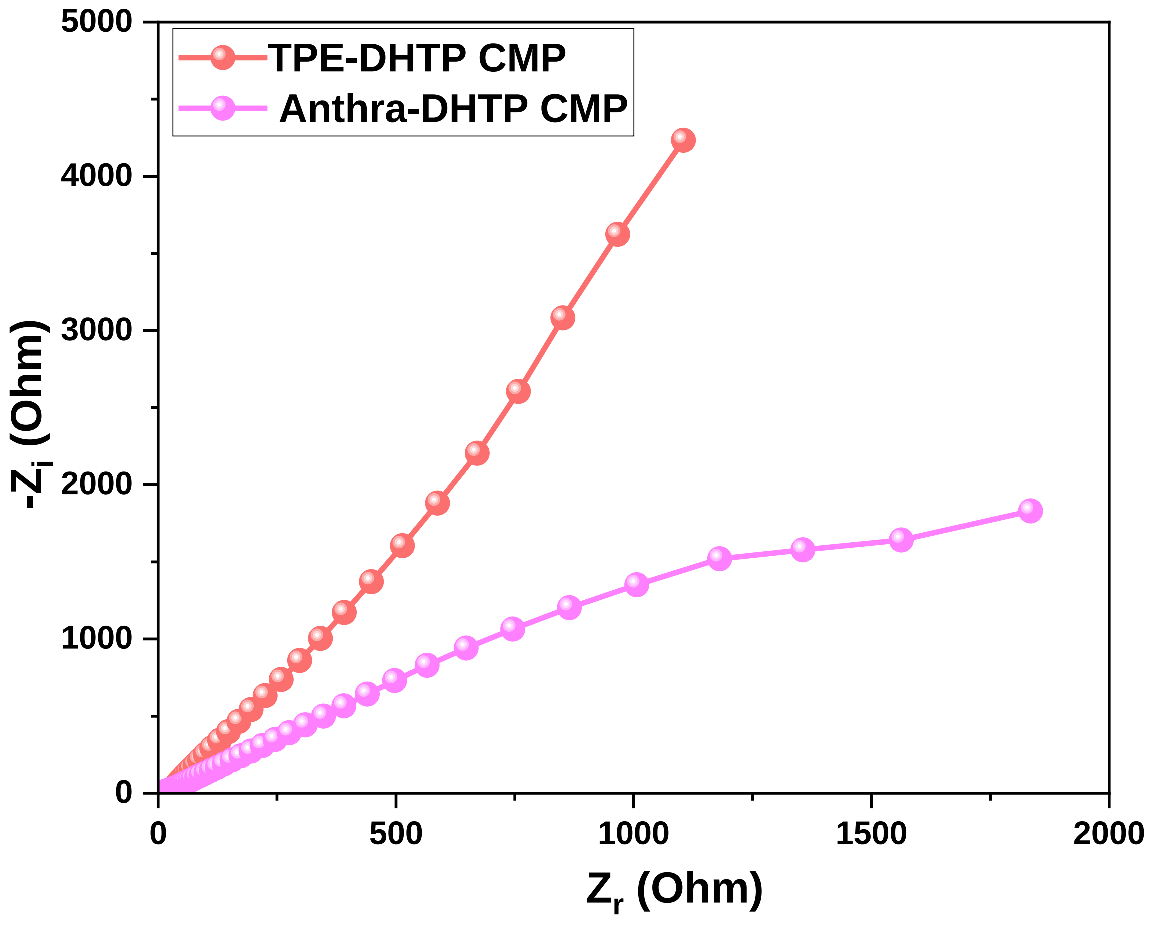

Figure S13. The EIS spectra of TPE-DHTP CMP and Anthra-DHTP CMP were measured after 5000 cycles.

Table S1. The specific capacitance of TPE-DHTP CMP derived from CV profiles was measured at various scan rates (using a three-electrode system).

**Scan Rate** **(mV s^{-1})**	**Specific capacitance** **(C g^{-1})**
**5**	**30.1**
**10**	**18.2**
**30**	**10.41**
**50**	**6.91**
**70**	**6.3**
**100**	**5.8**
**200**	**4.5**

Table S2. The specific capacitance of Anthra-DHTP CMP derived from CV profiles was measured at various scan rates (using a three-electrode system).

**Scan Rate** **(mV s^{-1})**	**Specific capacitance** **(C g^{-1})**
**5**	**70**
**10**	**36.34**
**30**	**28**
**50**	**25.1**
**70**	**23.51**
**100**	**22.83**
**200**	**18.8**

Table S3. Comparison of the TPE-DHTP CMP and Anthra-DHTP CMP supercapacitor performance with the electrodes that have been previously described.

Electrode	Capacitance	Ref.
**TPE-DHTP CMP**	**44 F g^{-1} at 0.5 A g^{-1}**	**This work**
**Anthra-DHTP CMP**	**121 F g^{-1} at 0.5 A g^{-1}**	**This work**
**Cz-Cz CMP**	43.70 F g^{-1} at 0.5 A g^{-1}	1
**Cz-TP CMP**	67.38 F g^{-1} at 1 A g^{-1}	1
**Pure AQ**	42 F g^{-1} at 1 A g^{-1}	2
**TPE-DDSQ-POIP**	22 F g^{-1} at 1 A g^{-1}	3
**Car-DDSQ-POIP**	23 F g^{-1} at 1 A g^{-1}	3
**Py-PDT POP**	28 F g^{-1} at 0.5 A g^{-1}	4
**TBN-BSU CMP**	70 F g^{-1} at 0.5 A g^{-1}	5
**Py-BSU CMP**	38 F g^{-1} at 0.5 A g^{-1}	5
**HPC-0**	48 F g^{-1} at 1 A g^{-1}	6
**H-THAQ**	15 F g^{-1} at 1 A g^{-1}	7
**CoPc-CMP**	13.8 F g^{-1} at 1 A g^{-1}	8
**β -Ketoenamine-Linked Covalent Organic Frameworks (COF)**	48 F g^{-1} at 0. 1 A g^{-1}	9
**TBN-Car-CMP**	18.45 F g^{-1} at 0.5 A g^{-1}	10
**TPE-Ph-Th CMP**	39 F g^{-1} at 0.5 A g^{-1}	11
**TPE-Ph-Tha CMP**	51 F g^{-1} at 0.5 A g^{-1}	11
**TPE-Ph-BSu CMP**	52 F g^{-1} at 0.5 A g^{-1}	11
**TBN-Diyne CMP**	39 F g^{-1} at 0.5 A g^{-1}	12
**TPE-Diyne CMP**	32.4 F g^{-1} at 0.5 A g^{-1}	12
**TPET-TTh CMP**	74 F g^{-1} at 0.5 A g^{-1}	13
**PyT-TTh CMPs**	76 F g^{-1} at 0.5 A g^{-1}	13

Table S4. Fitted EIS data of Anthra-DHTP CMP and TPE-DHTP CMP with characteristic values.

Sample	R_s	R_{ct}	CPE-EDL	CPE-P
**Anthra-DHTP CMP**	**20.9**	**184**	**4.4×10^{-5}**	**0.85**
**TPE-DHTP CMP**	**30.7**	**524**	**5.3×10^{-5}**	**0.9**

References

[1] Saber, S. F.; Sharma, S. U.; Lee, J. T.; EL-Mahdy, A. F. M.; Kuo, S. W. Carbazole-conjugated microporous polymers from Suzuki–Miyaura coupling for supercapacitors. *Polymer* **2022**, *254*, 125070. doi.org/10.1016/j.polymer.2022.125070.

[2] Guo, B.; Yang, Y.; Hu, Z.; An, Y.; Zhang, Q.; Yang, X.; Wang, X.; Wu, H. Redox-active organic molecules functionalized nitrogen-doped porous carbon derived from metal-organic framework as electrode materials for supercapacitor. *Electrochim. Acta*, **2017**, *223*, 74–84. doi.org/10.1016/j.electacta.2016.12.012.

[3] Mohamed, M. G.; Chen, W. C.; EL-Mahdy, A. F. M.; Kuo, S. W. Porous organic/inorganic polymers based on double- decker silsesquioxane for high- performance energy storage. *J. Polym. Res*. **2021**, *28*, 219. doi.org/10.1007/s10965-021-02579-x.

[4] Mousa, A. O.; Mohamed, M. G.; Chuang, C. H.; Kuo, S. W. Carbonized Aminal-Linked Porous Organic Polymers Containing Pyrene and Triazine Units for Gas Uptake and Energy Storage. *Polymers* **2023**, *15*, 1891. doi.org/10.3390/polym15081891.

[5] Mohamed, M. G.; Chang, S. Y.; Ejaz, M.; Samy, M. M.; Mousa, A. O.; Kuo. S. W. Design and Synthesis of Bisulfone-Linked Two-Dimensional Conjugated Microporous Polymers for CO_2 Adsorption and Energy Storage. *Molecules* **2023**, *28*, 3234. doi.org/10.3390/molecules28073234.

[6] Wan, L.; Wang, J.; Xie, L.; Sun, Y.; Li, K. Nitrogen-enriched hierarchically porous carbons prepared from polybenzoxazine for high-performance supercapacitors. *ACS Appl. Mater. Interfaces*, **2014**, *6*, 15583–15596. DOI: 10.1021/am504564q.

[7] Xu, L.; Shi, R.; Li, H.; Han, C.; Wu, M.; Wong, C. P.; Kang, F.; Li, B. Pseudocapacitive anthraquinone modified with reduced graphene oxide for flexible symmetric all-solid-state supercapacitors. *Carbon* **2018**, *127*, 459-68. DOI: 10.1016/j.carbon.2017.11.003.

[8] Mei, L.; Cui, X.; Duan, Q.; Li, Y.; Lv, X.; Wang, H. G. Metal Phthalocyanine-Linked Conjugated Microporous Polymer Hybridized with Carbon Nanotubes as a High-Performance Flexible Electrode for Supercapacitors. *Int. J. Hydrogen Energy* **2020**, *45*, 22950-22958. doi.org/10.1016/j.ijhydene.2020.06.208.

[9] DeBlase, C. R.; Silberstein, K. E.; Truong, T. T.; Abruña, D. H.; Dichtel, W. R. β-Ketoenamine-Linked Covalent Organic Frameworks Capable of Pseudocapacitive Energy Storage. *J. Am. Chem. Soc*. **2013**, *135*, 16821. doi.org/10.1021/ja409421d.

[10] Samy, M. M.; Mohamed, M. G.; El-Mahdy, A. F. M.; Mansoure, T. H.; Wu, W. C. W.; Kuo, S. W. High-Performance Supercapacitor Electrodes Prepared From Dispersions of Tetrabenzonaphthalene-Based Conjugated Microporous Polymers and Carbon Nanotubes. *ACS Appl. Mater. Interfaces* **2021**, *13*, 51906–51916. doi.org/10.1021/acsami.1c05720.

[11] Mohamed, M. G.; Hu, H. Y.; Santhoshkumar, S.; Madhu, M.; Mansoure, T. H.; Hsiao, C. W.; Ye, Y.; Huang, C. W.; Tseng, W. L.; Kuo, S. W. Design and Synthesis of Bifunctional Conjugated Microporous Polymers Containing Tetraphenylethene and Bisulfone Units for Energy Storage and Fluorescent Sensing of p-Nitrophenol. *Colloids Surf. A Physicochem. Eng. Asp*. **2024**, *680*, 132675. doi.org/10.1016/j.colsurfa.2023.132675.

[12] Mohamed, M. G.; Sharma, S. U.; Wang, P. T.; Ibrahim, M.; Lin, M. H.; Liu, C. L.; Ejaz, M.; Yen, H. J.; Kuo, S. W. Construction of Fully π-Conjugated, Diyne-Linked Conjugated Microporous Polymers Based on Tetraphenylethene and Dibenzo[*g,p*]chrysene Units for Energy Storage. *Polym. Chem*. **2024**. doi.org/10.1039/D4PY00421C.

[13] Sharma, S. U.; Elsayed, M. H.; Mekhemer, I. M. A.; Meng, T. S.; Chou, H. H.; Kuo, S. W.; Mohamed, M. G. Rational design of pyrene and thienyltriazine-based conjugated microporous

polymers for high-performance energy storage and visible-light photocatalytic hydrogen evolution from water. *Giant* **2024**, *17*, 100217. doi.org/10.1016/j.giant.2023.100217.
